# Development of a New Eco-Friendly Copolymer Based on Chitosan for Enhanced Removal of Pb and Cd from Water

**DOI:** 10.3390/polym14183735

**Published:** 2022-09-07

**Authors:** Iolanda-Veronica Ganea, Alexandrina Nan, Carmen Roba, Iulia Neamțiu, Eugen Gurzău, Rodica Turcu, Xenia Filip, Călin Baciu

**Affiliations:** 1 Faculty of Environmental Science and Engineering, Babes-Bolyai University, 30 Fantanele, 400294 Cluj-Napoca, Romania; 2 Development of Isotopic and Molecular Technologies, National Institute for Research, 67-103 Donath, 400293 Cluj-Napoca, Romania; 3 Environmental Health Center, 58 Busuiocului, 400240 Cluj-Napoca, Romania; 4 Cluj School of Public Health, College of Political, Administrative and Communication Sciences, Babeș-Bolyai University, 7 Pandurilor, 400095 Cluj-Napoca, Romania

**Keywords:** eco-friendly copolymer, poly(benzofurane-*co*-arylacetic acid), chitosan, heavy metals removal, wastewater, Roșia Montană, adsorption mechanism

## Abstract

Worldwide, concerns about heavy metal contamination from manmade and natural sources have increased in recent decades. Metals released into the environment threaten human health, mostly due to their integration into the food chain and persistence. Nature offers a large range of materials with different functionalities, providing also a source of inspiration for scientists working in the field of material synthesis. In the current study, a new type of copolymer is introduced, which was synthesized for the first time by combining chitosan and poly(benzofurane-*co*-arylacetic acid), for use in the adsorption of toxic heavy metals. Such naturally derived materials can be easily and inexpensively synthesized and separated by simple filtration, thus becoming an attractive alternative solution for wastewater treatment. The new copolymer was investigated by solid-state nuclear magnetic resonance, thermogravimetric analysis, scanning electron microscopy, Fourier transform infrared spectroscopy, and X-ray photon electron microscopy. Flame atomic absorption spectrometry was utilized to measure heavy metal concentrations in the investigated samples. Equilibrium isotherms, kinetic 3D models, and artificial neural networks were applied to the experimental data to characterize the adsorption process. Additional adsorption experiments were performed using metal-contaminated water samples collected in two seasons (summer and winter) from two former mining areas in Romania (Roșia Montană and Novăț-Borșa). The results demonstrated high (51–97%) adsorption efficiency for Pb and excellent (95–100%) for Cd, after testing on stock solutions and contaminated water samples. The recyclability study of the copolymer indicated that the removal efficiency decreased to 89% for Pb and 58% for Cd after seven adsorption–desorption cycles.

## 1. Introduction

It is well-known that anthropogenic impact causes water pollution, habitat loss or degradation, and spread of invasive species, thus affecting marine ecosystems, wildlife, and human health, and contributing to climate change and quantitative as well as qualitative decrease of freshwater resources [1,2,3]. These effects are harmful not only to individual species and populations but also to entire communities [4,5,6]. Nowadays, heavy metal pollution has become a serious problem due to metals’ difficult natural degradation processes and persistence in the environment, from where they are gradually released into water bodies which serve as sinks for contaminant discharge [7]. Moreover, human exposure to toxic concentrations of cadmium and lead can cause acute symptoms (e.g., irritation, abdominal pain, diarrhea, headache, nausea) and long-term effects (e.g., “itai-itai” disease, renal tubular dysfunction, encephalopathy, hypertensive disorders, cancer, coma, and death) [8,9].

Nowadays, scientists find inspiration from the enormous variety of materials that nature offers and their diverse uses. Adsorbents derived from biopolymers, including polysaccharides, have the benefits of being biocompatible and biodegradable [10]. For instance, chitosan is a polysaccharide synthesized through the deacetylation of chitin (the second most abundant polymer in nature after cellulose) and is used in a variety of fields including catalysis, biomedicine, veterinary medicine, pharmaceuticals, drug delivery, decontamination, membrane and film synthesis, food science, and enzyme immobilization [11,12,13,14]. Chitin can be found in significant quantities in seafood processing waste produced in many eastern and southeastern Asian countries [15]. Compared with conventional adsorbents, chitosan is biocompatible, antibacterial, biodegradable, easily separable through filtration, involves low costs, and has amino and hydroxyl functional groups that provide effective binding sites for contaminants, especially heavy metals (through chelation, i.e., ion exchange) [16]. The nitrogen atom in amino groups is the donor of electrons, while metal ions act as acceptors [17]. Chemical or physical modifications can improve chitosan’s poor solubility and small surface area. Physical modification methods can enable processing into membranes, beads, nanofibers, gels, nanoparticles, honeycomb, etc. [18,19]. The common chemical modifications that can be applied to chitosan include N-alkylation, acylation, carboxylation, esterification using inorganic oxygen acids or anhydrides, grafting on polymers such as poly(ethylene imine), polyaniline, poly(vinyl amine), poly(alkyl methacrylate), poly(vinyl alcohol), triethylenetetramine, or polyacrylamide, crosslinking with glyoxal, tripolyphosphate, ethylene glycol diglycidyl ether, formaldehyde, epichlorohydrin, glutaraldehyde, dimethyloldihydroxy ethylene urea, or isocyanates, etc. [20,21,22,23,24,25,26,27,28,29,30,31,32,33]. Several studies have reported that pure chitosan has affinity for metals in the following order: Hg > Cu > Fe > Ni > Ag > Cd > Mn > Pb > Co > Cr, while others have stated that after cross-linking, this turns to Cu > Pb > Zn [34,35,36,37,38].

The present study introduces a new hybrid material, **CHIT-PAAA**, which was synthesized through the modification of chitosan (**CHIT**) with poly(benzofurane-*co*-arylacetic acid) (**PBAAA**), using simple green chemical methods. The obtained copolymer was structurally and morphologically investigated by solid-state nuclear magnetic resonance (ss-NMR), scanning electron microscopy (SEM), X-ray photon electron spectroscopy (XPS), and Fourier transform infrared spectroscopy (FTIR). Thermogravimetric analysis (TGA) was used to evaluate the material’s thermal stability. Furthermore, the material was put to use to remove heavy metals from contaminated water samples. Flame atomic absorption spectrometry (FAAS) measurements were performed to determine the metal concentrations. A preliminary adsorption study was conducted to check the suitability of this material for environmental applications [39]. Pb and Cd were chosen for applying various isotherms and kinetic models, and to observe the adsorption behavior of the material based on the initial contaminant concentration and contact time. Furthermore, 3D adsorption rate models and artificial neural networks (ANNs) were also generated to characterize the adsorption process.

## 2. Materials and Methods

### 2.1. Chemical Reagents

**PBAAA** was synthesized by closely following a previously reported procedure [40], being highly soluble in most commonly used solvents. Chitosan (medium molecular weight), cadmium chloride hydrate 98% (CdCl_2_ × H_2_O), and lead chloride 98% (PbCl_2_) were purchased from Sigma-Aldrich (St. Louis, MO, USA). All reagents used were of analytical grade, commercially available, and no further purification was involved.

### 2.2. Synthesis of the Adsorbent Material

**Scheme 1:**
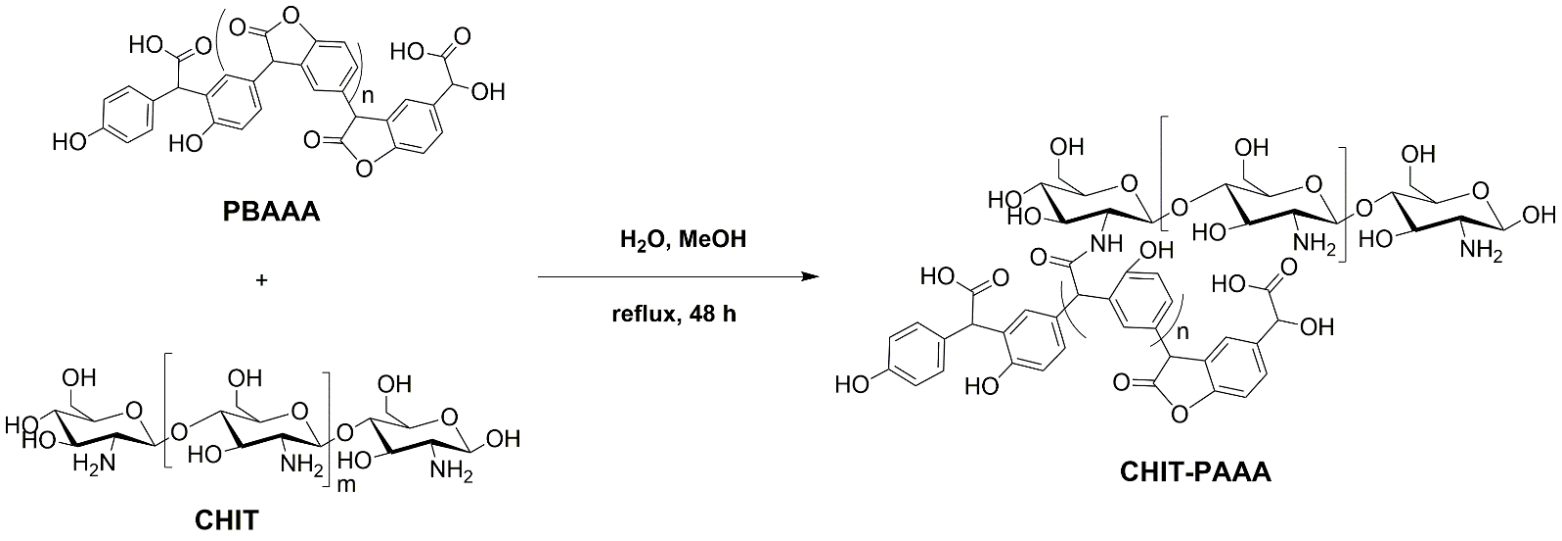
Synthesis of **CHIT-PAAA**.

Preparation of the new copolymer through the modification of chitosan with **PBAAA** is shown in Scheme 1. **PBAAA** (2 g) and chitosan (1 g) were placed in a 250 mL flask and dissolved in a mixed solution of deionized water (150 mL) and methanol (20 mL). The prepared solution was ultrasonicated for 1 h and then refluxed for 2 days. After the reaction finished, the solvents were evaporated using a rotary evaporator. Furthermore, a mixture of methanol and deionized water (2:1) was added to the remaining solid, and the solution was ultrasonicated for 30 min. The solid material was filtered off and subsequently washed with methanol. Finally, the product **CHIT-PAAA** was dried at room temperature and then analyzed. The homopolymer was removed with the water–methanol mixture to evaluate the efficiency of the graft copolymerization. Although there are no unified definitions for calculating the parameters of the graft copolymerization, herein we report the use of the grafting yield (G) and the copolymerization yield (Y) (Equations (1) and (2)) [41]:

(1) $$G\left(\%\right)=\frac{W_{CHIT-PAAA}-W_{CHIT}}{W_{CHIT}}\;\cdot 100$$

(2) $$Y\;\left(\%\right)=\frac{W_{CHIT-PAAA}}{W_{CHIT}+W_{PBAAA}}\;\cdot 100$$

where W**_CHIT-PAAA_** is the mass of the copolymer after grafting, W**_CHIT_** is the initial weight of chitosan added in the copolymerization reaction, and W**_PBAAA_** is the initial weight of **PBAAA** added in the copolymerization reaction.

### 2.3. Characterization Methods

#### 2.3.1. Solid-State Nuclear Magnetic Resonance

A Bruker Advance III 500 MHz wide-bore NMR spectrometer operating at room temperature was used, with a 4 mm double resonance (1H/X) MAS probe. The material was packed in 4 mm zirconia rotors, and the solid-state ^13^C and ^15^N NMR spectra were recorded at 125.73 and 50.66 MHz Larmor frequencies. Standard RAMP ^13^C/^15^N CP-MAS spectra were acquired at 14/7 kHz spinning frequencies, 2/4 ms contact times, and proton decoupling under TPPM. For ^13^C spectra, the acquisition parameters were optimized to the following values of relaxation delay and number of transients: 2 s/30,000 transients for **PBAAA** and **CHIT**, and 2 s/50,000 transients for sample **CHIT-PAAA**. For ^15^N spectra the relaxation delay and number of transients were: 2 s/31,000 transients for **CHIT** and 2 s/60,000 transients for **CHIT-PAAA**. The recorded spectra were calibrated relative to the CH_3_ line in tetramethylsilane (TMS) and the ^15^NO_2_ line in nitromethane, through an indirect procedure that used L-Glycine as an external reference (C=O of glycine at 176.5 ppm for ^13^C and −347.6 ppm for ^15^N), and line broadening was applied at 20 Hz (for ^13^C spectra) and 150 Hz (for ^14^N spectra).

#### 2.3.2. Fourier Transform Infrared Spectroscopy

FTIR investigation was conducted on a Jasco FTIR-6100 spectrophotometer (JASCO Deutschland GmbH, Pfungstadt, Germany), recording the material’s spectra in the 400–4000 cm^−1^ spectral range. Pressed pellets prepared from polymer powder embedded in KBr were used for this purpose.

#### 2.3.3. Scanning Electron Microscopy

SEM analysis was performed on a Hitachi SU8230 High-Resolution Scanning Electron Microscope (Hitachi Ltd., Tokyo, Japan) equipped with a cold field-emission gun. The samples were placed on aluminum stubs and covered with a 10 nm gold coating for morphological analysis.

#### 2.3.4. Thermo-Gravimetric Analysis

TGA was conducted in air, using TA Instruments SDT Q 600 equipment (TA Instruments Inc., New Castle, DE, USA), in the temperature range 30 °C–800 °C, with a heating rate of 10 °C min^−1^ in air.

#### 2.3.5. X-ray Photon Electron Spectroscopy

An XPS spectrometer SPECS (SPECS Surface Nano Analysis GmbH, Berlin, Germany) equipped with a dual-anode X-ray source Al/Mg, a PHOIBOS 150 2D CCD hemispherical energy analyzer, and a multi-channeltron detector with vacuum maintained at 1 × 10^−9^ torr was used to record XPS spectra. XPS investigations were conducted using the Al Kα X-ray source (1486.6 eV) operating at 200 W. The XPS survey spectra were captured at 30 eV pass energy, 0.5 eV/step. The high-resolution spectra for individual elements were recorded by accumulating 30 scans at 30 eV pass energy and 0.1 eV/step. The powder samples were pressed on an indium foil to allow the XPS measurements. The sample surface was cleaned by argon ion bombardment (300 V) and the spectra were recorded before and after the cleaning. Data analysis and curve fitting were performed using CasaXPS software (Casa Software Ltd., Teignmouth, UK) with Gaussian-Lorentzian product functions and a non-linear Shirley background subtraction.

#### 2.3.6. Brunauer-Emmett-Teller Surface Area Analysis

The total surface area (St), pore volume (Vp), and pore radius (Rm) of N_2_ adsorption–desorption isotherms (recorded at −196 °C) were determined using the Brunauer–Emmett–Teller (BET) technique for measuring St, and the Dollimore–Heal model. A Sorptomatic 1990 device (Thermo Electron Corporation, Waltham, MA, USA) was used to record the isotherms. Prior to analysis, samples were degassed at 70 °C for 5 h at a pressure of 1 Pa to eliminate any physisorbed contaminants from the surface.

#### 2.3.7. Flame Atomic Absorption Spectrometry

FAAS was used in batch experiments to determine the heavy metal concentrations. The samples were atomized using an atomic absorption spectrophotometer AAS Spectra AA110 (Varian, Australia) in a flame of air and acetylene. The analysis method closely followed the protocol described in detail in the standard SR ISO 8288/2001. In brief, the samples were digested in nitric acid, and five-point calibration curves were drawn for each metal, with the range of concentrations between 0.05 mg L^−1^ and 0.4 mg L^−1^ for Cd, and between 0.25 mg L^−1^ and 2.50 mg L^−1^ for Pb. Dilutions were made for samples that had concentrations exceeding the intervals previously mentioned. The reference material used for standard preparation (for the calibration curves) was 1000 mg L^−1^ Spectro Econ Chem Lab Stock Solution (Chem Lab, Zedelgem, Belgium), while 1000 mg L^−1^ Merck Stock Solution (Merck KGaA, Darmstadt, Germany) was used for quality control. The method’s detection limits were 0.03 mg L^−1^ for Cd and 0.25 mg L^−1^ for Pb.

### 2.4. Batch Adsorption Experiments

Stock solutions of metal contaminants (Pb, Cd) were prepared at six different concentrations (10, 20, 40, 60, 80, and 100 mg L^−1^) using Pb and Cd salts (PbCl_2_, CdCl_2_ × H_2_O) and Milli-Q ultrapure water (Millipore, Bedford, MA, USA) with pH adjusted to 5.0. The effects of two parameters (initial metal concentration and contact time) were investigated to study the adsorptive behavior of **CHIT-PAAA**. Pb and Cd adsorption assays were performed on the synthesized material **CHIT-PAAA** under magnetic agitation (at 600 rpm rotational speed) and normal atmospheric conditions (room temperature). Afterwards, samples were filtered off (on Rotilabo folded filters, type 113 P, membrane Ø 150 mm, Macherey-Nagel GmbH, Dueren, Germany), and heavy metals in the supernatant were analyzed by FAAS. An AAS Spectra AA110 atomic absorption spectrophotometer was used to determine the metal concentrations in the solutions.

The removal efficiencies (adsorption percentages) and sorption capacities were calculated based on the following equations:

(3) $$R\;\left(\%\right)=\frac{C_{i}-C_{f}}{C_{i}}\cdot \;100$$

(4) $$q\;\left({\mathrm{mg}\;g}^{-1}\right)=\frac{\left(C_{i}-C_{f}\right)\cdot \;V}{w}\;$$

where R is the removal efficiency (%); C_i_ is the initial concentration (before adsorption) (mg L^−1^); C_f_ is the final concentration (after adsorption) (mg L^−1^); q is the sorption capacity (mg g^−1^); V is the volume of solution (L); w is the amount of sorbent (material) used (g).

### 2.5. Equilibrium Adsorption Isotherms

Pb and Cd adsorption equilibrium studies were carried out using 0.04 L metal stock solutions of six different concentrations (10, 20, 40, 60, 80, and 100 mg L^−1^) and 0.02 g adsorbent material for 24 h contact time. Linear and nonlinear forms of Langmuir [42], Freundlich [43], Dubinin–Radushkevich [44], Temkin [45], Khan [46], Redlich–Peterson [47], Sips [48], Toth [49] and Koble–Corrigan [50] isotherm models were applied to fit the **CHIT-PAAA** experimental adsorption data. Table S1 summarizes the equations of the isotherms used in the current study. For the linear forms, the values of each isotherm constant were obtained from the slope and intercept of various plots: C_e_/q_e_ versus C_e_ (Langmuir 1st type), ln(q_e_) versus ln(C_e_) (Freundlich), ln(q_e_) versus ε^2^ (Dubinin–Radushkevich), q_e_ versus ln(C_e_) (Temkin), ln(C_e_/q_e_) versus ln(C_e_) (Redlich–Peterson), ln(q_e_)/(q_max_−q_e_)) versus ln(C_e_) (Sips).

The separation factor was also determined, because it highlights the essential characteristics of Langmuir isotherm (the shape of the isotherm and the nature of the adsorption process):

(5) $$R_{L}=\frac{1}{1+K_{L}\;C_{i}}\;$$

where $R_{L}$ is the separation factor, $C_{i}$ is the initial concentration of the metal ion solution (mg L^−1^), and $K_{L}$ is the Langmuir constant (L mg^−1^). The nature of the adsorption process can be categorized as unfavourable ($R_{L}$ > 1), linear ($R_{L}$ = 1), favourable (0 < $R_{L}$ < 1), or irreversible ($R_{L}$ = 0) [51,52].

### 2.6. Kinetic Studies

The prediction of batch adsorption kinetics was essential to describe the adsorption rates and sorbate interactions. The **CHIT-PAAA** kinetic experiments were conducted using 0.04 g adsorbent material and 0.08 L metal solutions of 10, 20, 40, 60, 80 and 100 mg L^−1^ concentrations. Samples were collected from each solution after 1 min, 5 min, 10 min, 20 min, 30 min, 45 min, 1 h, 3 h, 6 h, 9 h, 12 h, and 24 h of contact time, and heavy metals were determined via FAAS. Four types of kinetic models were applied to characterize the adsorption behaviour of **CHIT-PAAA**, namely the pseudo-first order [53], pseudo-second order [54], Weber–Morris intra-particle diffusion [55], and Elovich model [56]. Table S2 summarizes the equations of the models used to determine the adsorption kinetics of Pb and Cd onto **CHIT-PAAA**. Another useful kinetic parameter is the adsorption half-time (τ12) which represents the amount of time needed to attain half the adsorption progress or half the equilibrium value [57,58]. This parameter can be calculated as follows:

(6) τ12[min]=1k2· qe

### 2.7. Statistics

The results of all equilibrium and kinetic models used in this study were evaluated through the least-square method and correlation coefficient (R^2^) analysis. The statistical evaluation was performed using Origin v.2018 (OriginLab Corporation, Northampton, MA, USA). The root mean square error function was also determined in order to establish the best-fitting models [59]:

(7) $$\mathrm{RMSE}\;=\sqrt{\frac{1}{n}\cdot \;\sum_{i=1}^{n}{\left(q_{\mathrm{calc}\left(i\right)}-{\;q}_{\exp\left(i\right)}\right)}^{2}}$$

where RMSE represents the root mean square error; q_calc_ is the calculated amount of pollutant adsorbed per unit mass of material (mg g^−1^); q_exp_ is the measured amount of pollutant adsorbed per unit mass of material (mg g^−1^).

### 2.8. Recyclability Studies

Seven adsorption–desorption cycles were conducted to check the reusability of **CHIT-PAAA**. For this purpose, 10 mg L^−1^ Pb and Cd aqueous solutions were shaken at 600 rpm for 1 h with specific amounts of copolymer. Afterwards, the material was separated from the contaminated solutions through filtration and subsequently washed with 30 mL 0.1 M HNO_3_ solution and distilled water. The filtrated solutions were analysed using FAAS.

### 2.9. 3D Adsorption Rate Models

A 3D adsorption rate model is a representation providing a clear overview of the adsorption process and the factors that influence the sorption capacities of a studied material [60]. Herein, contact time and initial metal concentration were considered the main parameters affecting **CHIT-PAAA** adsorption rates. The 3D adsorption rate models were generated with a resolution of 1 mg L^−1^, over a range of initial metal concentrations from 1 to 100 mg L^−1^ for each pollutant.

### 2.10. Artificial Neural Networks Models

The high complexity of the adsorption process makes it difficult to model only through statistical methods. Therefore, computational intelligence models such as adaptive fuzzy inference systems (ANFIS), least square support vector regression (LSSVR), random forest (RF), or artificial neural networks (ANNs), which rely on artificial intelligence (AI) prediction, represent some of the best methods for modeling complex datasets [61,62,63,64,65,66,67,68]. ANNs were first introduced by McCulloch and Pitts [69], inspired by the structure and functions of biological neural networks, and have become a powerful tool for predicting system behaviors and for analyzing processes [69,70,71,72]. In general, ANNs consist of artificial neurons with specific weights, placed in various layers, interconnected through a system of artificial synapses that train interrelationships between inputs and outputs [73].

A multilayer perceptron (MLP), one of the most common types of ANNs, includes an input layer, an output layer, and one or more hidden (intermediate) layers. The numbers of layers and neurons, the networks’ structure, the transfer function, and the training component form the architecture of an ANN [74]. ANNs undergo a training algorithm to enable them to predict the correlation between inputs and outputs and to reproduce known and unknown data. The MLP network is a feed-forward ANN, because data are processed from the input to the output layers [60]. The most common ANN structures used for adsorption experiments are multilayer feed-forward neural networks (MLFFN) [75]. Collected data is usually divided into 70–80% training data (for generating the output values) and 20–30% testing data (for examining the parameters of the trained ANN). The performance of the ANN model can be checked and improved by adjusting the mean squared error function (MSE) and the correlation coefficient defined by the following equations [76]:

(8) $$\mathrm{MSE}\;=\frac{1}{n}\;\sum_{i=1}^{n}{\left(\left|{\hat{Y}}_{i}-Y\right|\right)}^{2}$$

(9) $$R^{2}=1-\frac{\sum_{i=1}^{n}\left({\hat{Y}}_{i}-Y\right)}{\sum_{i=1}^{n}\left({\hat{Y}}_{i}-Y_{\mathrm{av}}\right)}$$

where n is the number of data, $\hat{Y}$ represents the predicted data, Y is the actual output data, and Y_av_ is the average of the experimental values.

For the current study, a three-layer ANN (two inputs and one output) was developed by using the Neural Network Toolbox of MATLAB 7.6 (R2008a) mathematical software (MathWorks, Natick, MA, USA). The three layers consisted of two neurons in the input layer represented by the initial metal concentration (10, 20, 40, 60, 80, or 100 mg L^−1^) and contact time (0–1440 min), and one neuron in the output layer (the amount of metal adsorbed). The ANN was trained with 840 data points and validated with 180. Algorithms involved 1000 iterations with tangent sigmoid transfer functions (*tansig*) and linear transfer functions (*purelin*) for training the MLFFN.

### 2.11. Adsorption Assays on Metal-Polluted Water Samples

**Figure 1:**
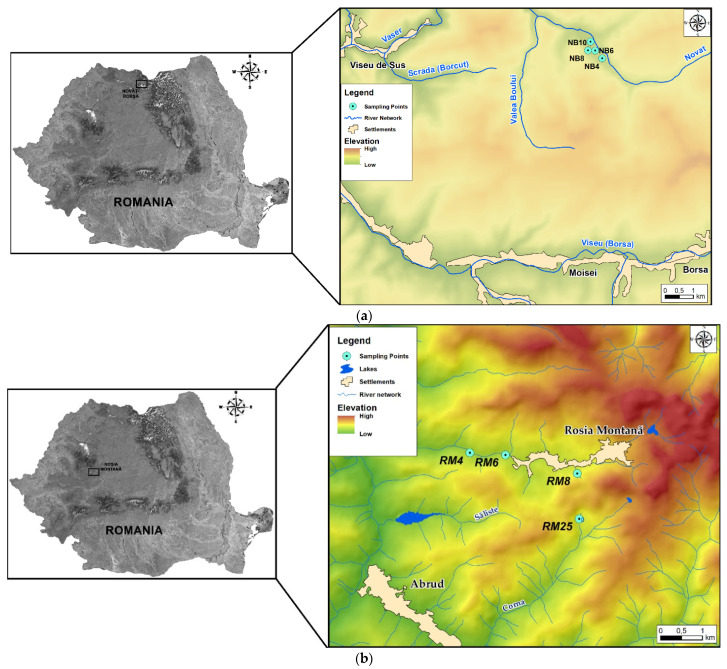
Map of the collected water samples: (**a**) Novăț-Borșa mining area, (**b**) Roșia Montană mining area.

Four water samples (Table S3) were collected in two seasons (summer and winter 2020) from two former mining areas in Romania (Novăț-Borșa and Roșia Montană, Figure 1). The map with the sampling points was developed using ArcGIS 10.6.1 (ESRI, Redlands, CA, USA). Novăț-Borșa is located in northern Romania, in the Maramureș Mountains (Maramureș County), close to the border with Ukraine. It has been an important source of lead and zinc ores (associated with copper, antimony, bismuth, cadmium, gold, and silver). Roșia Montană is situated in western Romania, in the Apuseni Mountains (Alba County) and has been exploited for its gold and silver ores. These two sampling areas were selected because they are considered among the most polluted sites in Romania, with soil, groundwater, and surface waters often reported to contain significant loads of heavy metals.

The **CHIT-PAAA** adsorption experiments using the collected metal-polluted water samples were carried out in similar conditions as the stock solution assays. After following the adsorption protocol, the metal content in the water samples was determined with FAAS.

## 3. Results and Discussion

### 3.1. Synthesis and Characterization of the Adsorbent Material

Graft polymerization represents one approach to the fabrication of chemically bonded natural–synthetic copolymer compositions. Grafting has been utilized as an important technique for modifying the chemical and physical properties of polymers. Graft copolymers are increasingly gaining importance due to their tremendous industrial potential. The current study explored the possibility of obtaining a new eco-friendly material, insoluble in water and easily separable via filtration, by opening the lactone rings in the **PBAAA** polymer chain [40] with the free amino groups of chitosan in an uncatalyzed reaction. The grafting yield determined for **CHIT-PAAA** copolymer was 140% and the copolymerization reaction yield was 83%.

**Figure 2:**
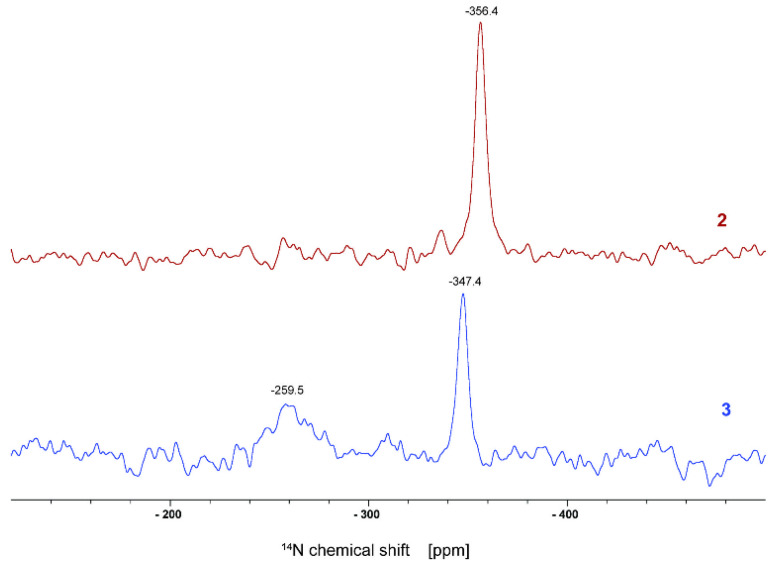
^15^N *ss*-NMR spectra of the: **CHIT** (red line) and **CHIT-PAAA** (blue line).

Considering the fact that **CHIT-PAAA** is solid and insoluble in water or organic solvents, NMR-spectra were recorded in solid state, i.e., as ^13^C *ss*-NMR and ^15^N *ss*-NMR spectra. As shown in Figure 2, there is only one signal in the ^15^N *ss*-NMR spectrum of chitosan, whereas two signals for nitrogen atoms can be observed in the spectrum of the final copolymer. The peak at −347.4 ppm of the copolymer is attributed to the −NH_2_ group of the chitosan chain, and the weaker broad peak at −259.5 ppm is assigned to the new amide bond (-NH-C=O), which appears after the covalent linkage of chitosan to the **PBAAA** by opening the lactone ring.

**Figure 3:**
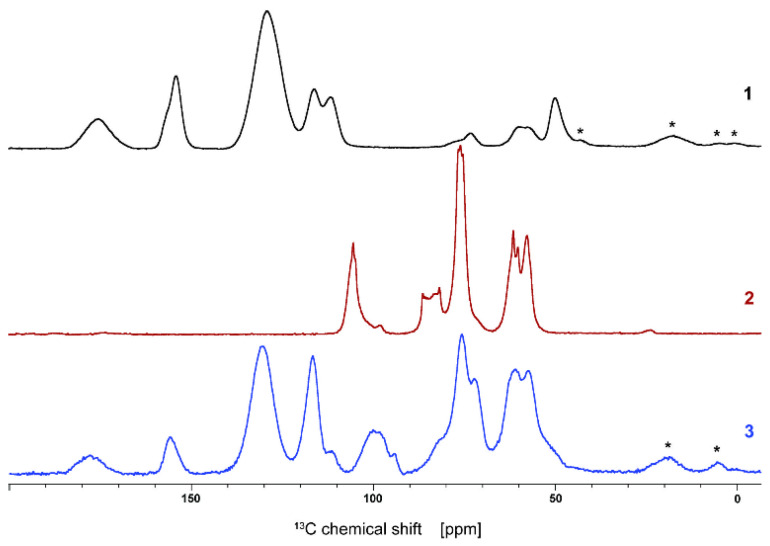
^13^C ss-NMR spectra of **PBAAA** (black), **CHIT** (dark red) and **CHIT-PAAA** (blue). The asterisk * indicates a sideband.

Significant changes were also seen in the ^13^C *ss*-NMR spectrum of the final copolymer compared with the starting materials (Figure 3). As a result of the attachment of the chitosan to the **PBAAA** chain, the peaks at around 50 ppm in the ^13^C *ss*-NMR spectra of **PBAAA,** typical for the –CH in the lactone units, do not appear in the ^13^C *ss*-NMR spectra of copolymer **CHIT-PAAA**. The peak in the 15–25 ppm region indicates -CH groups belonging to the chitosan chain. It was also present in the spectrum of the copolymer but was hidden under the sideband. Moreover, a significant additional change in the ^13^C *ss*-NMR spectrum of the copolymer was also observed in the aromatic part, where the signals for the peaks belonging to the benzene ring of **PBAAA** appeared due to the covalent linkage of the chitosan on the polymer chain.

**Figure 4:**
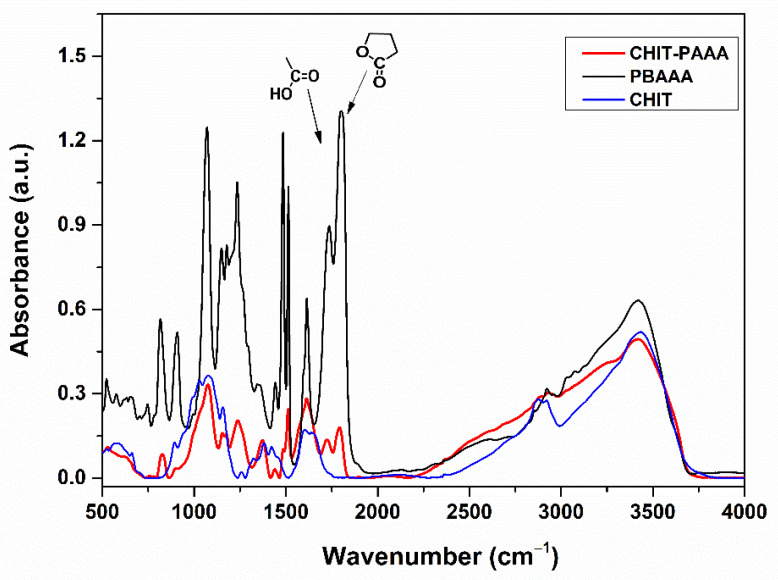
FTIR spectra of **PBAAA** (black), **chitosan** (blue), and copolymer **CHIT-PAAA** (red).

Figure 4 shows the FTIR spectra of **PBAAA, CHIT** and **CHIT-PAAA**. The latter contains both sets of bands of the starting materials **CHIT** and **PBAAA,** showing the linkage of both moieties. In addition, the absorption band around 1620 cm^−1^ belonging to the amide bond of **CHIT-PAAA** was more intensive than in the case of **CHIT**. This demonstrates the covalent attachment of the polymer chain to chitosan. Furthermore, a decrease in band intensity at 1800 cm^−1^ attributable to the lactone C=O bond was observed in comparison with the copolymer with r, indicating the opening of lactone rings. Also, this is due to the linkage of the amino group of chitosan to the lactone ring of r. The FTIR bands located between 1381–1457 cm^−1^ are attributed to the –C-H bond of the -CHOH-group, while those between 991–1078 cm^−1^ for the copolymer are typical for the –C-O-bond in the –COH group.

**Figure 5:**
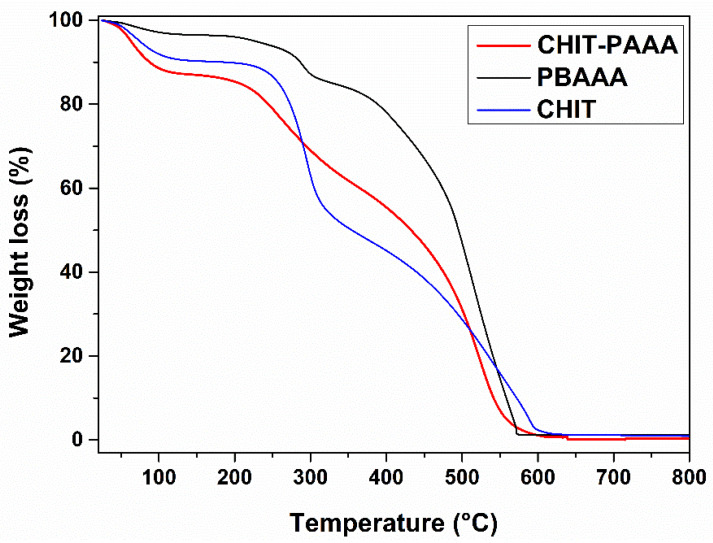
TGA curves of **PBAAA** (black), **CHIT** (blue) and **CHIT-PAAA** (red).

Figure 5 presents the TGA curves of **CHIT**, **PBAAA** and **CHIT-PAAA,** ranging from room temperature to 800 °C. **CHIT** underwent three degradation phases; first, between 39 °C–151°C (9.9% weight loss) corresponding to the elimination of water adsorbed in the polysaccharide structure; second, between 230 °C–360 °C (39% weight loss); and third, above 360 °C, attributed to total degradation. An increment in the decomposition trend of organic matter was observed in the case of **CHIT-PAAA** compared to **PBAAA**, due to the higher number of hydroxyl groups present after the covalent linkage of the polymer to the chitosan chain. Thus, an initial weight loss of 6.9% at 284 °C was recorded for **PBAAA**, associated with a decarboxylation process, followed by continuous degradation until 570 °C. Regarding the new material **CHIT-PAAA**, a 61% weight loss was observed in two steps; first, between 45 °C–105 °C (13% weight loss) and then between 200 °C–550 °C (48% weight loss), corresponding to the decomposition of the polysaccharide structure of the chitosan. The whole copolymer structure collapsed at 570 °C.

**Figure 6:**
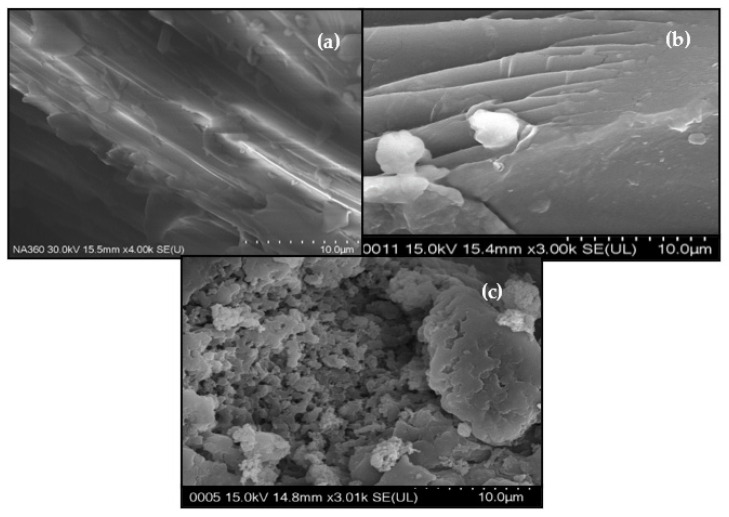
SEM images of (**a**) **PBAAA**, (**b**) **CHIT** and (**c**) **CHIT-PAAA**.

**PBAAA**, **CHIT**, and **CHIT-PAAA** were also investigated through SEM (Figure 6). Major morphological differences can be seen: **PBAAA** resembles an arboreal self-assembling structure, while **CHIT** has a flat, uniform folded surface, and **CHIT-PAAA** forms aggregates with a cauliflower-like aspect. The rough, heterogeneous surface of **CHIT-PAAA** with many pores makes it suitable as an adsorbent for different applications.

### 3.2. Metal Removal Assays on Stock Solutions

**Figure 7:**
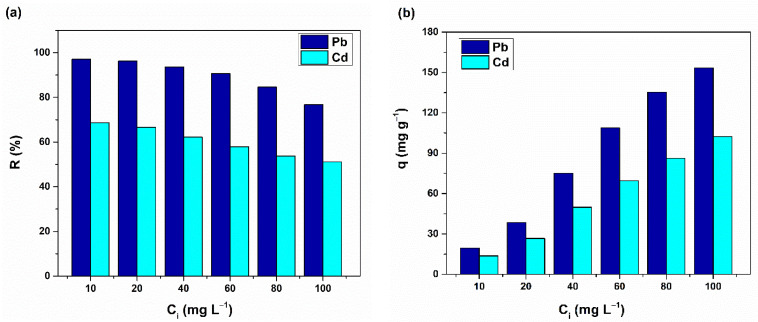
(**a**) Removal efficiency and (**b**) sorption capacity of **CHIT-PAAA** for Pb and Cd stock solutions.

The experiments on stock solutions started with investigation of the effects induced by contact time and Pb and Cd initial concentrations on the adsorption efficiency and sorption capacity of **CHIT-PAAA**. As indicated in Figure 7a, Pb recorded very high removal efficiencies (90.63–96.07%) for a range of metal concentrations between 10–60 mg L^−1^. However, a decreasing trend (76.71–84.60%) can be noticed at high concentrations (80–100 mg L^−1^). Cd also recorded better adsorption efficiencies (62.20–68.60%) at low concentrations, compared with those obtained with elevated concentrations. As expected, sorption capacity increased proportionally to metal concentration (153.42 mg g^−1^ and 102.26 mg g^−1^ were obtained at 100 mg L^−1^ for Pb and Cd, respectively; Figure 7b).

The Pb and Cd sorption capacities of the newly synthesized copolymer determined in the current study are comparable to the results reported in the literature for other chitosan-based materials, as listed in Table 1. **CHIT-PAAA** recorded excellent adsorption capacities for both investigated metals compared with most adsorbents tested by other researchers [77,78,79,80,81,82,83,84,85,86,87]. Moreover, the copolymer registered sorption capacities up to 18 times higher than CHIT, the parent component, as seen in Table 1.

The changes occurring in the **CHIT-PAAA** structure after Pb and Cd sorption could easily be noticed in the infrared spectra of the copolymer (Figure S1). Equal amounts of material were used to make the pellets used for FTIR determination, and all obtained spectra were normalized after recording. Important modifications emerged in the 350–600 cm^−1^ wavenumber range, namely the intensification of Pb-O, Cd-O bands at 462 cm^−1^ and 505 cm^−1^. Furthermore, other rises were seen in the bands belonging to C-O, C=C bonds from the benzene ring, and in the C-N, -N-H-, C=O bonds, located at 1072 cm^−1^, 1156 cm^−1^, 1245 cm^−1^, 1377–1435 cm^−1^, and 1513–1617 cm^−1^.

**Figure 8:**
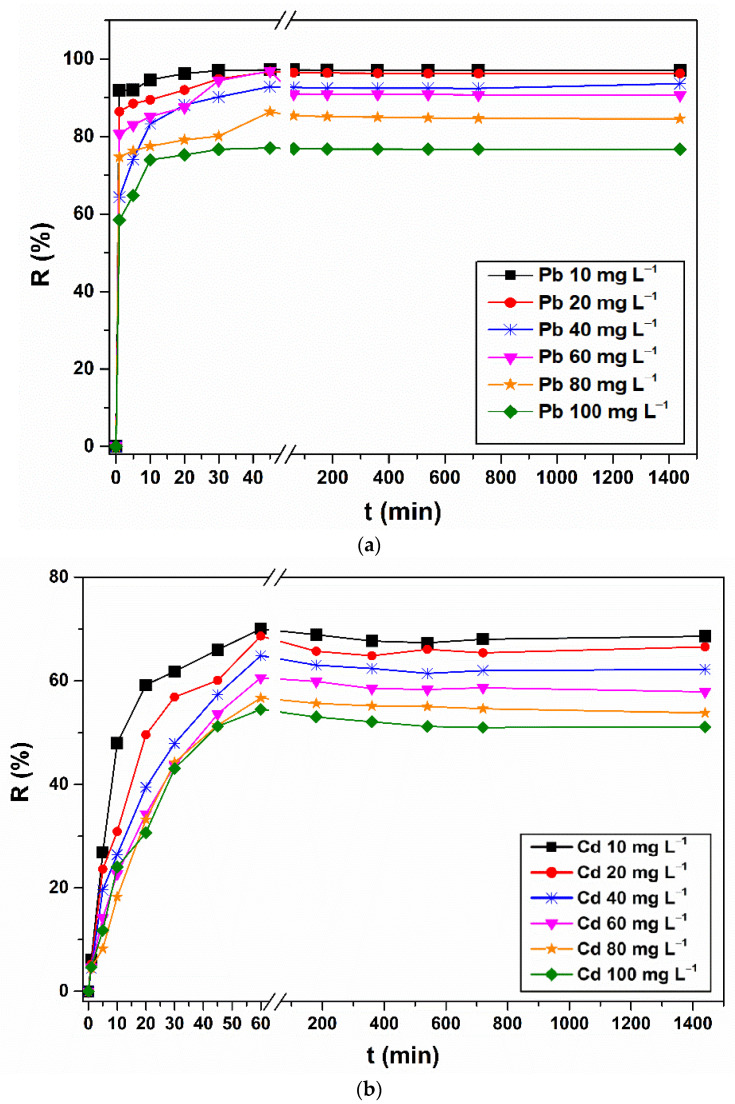
The effect of contact time on (**a**) Pb and (**b**) Cd removal efficiencies of **CHIT-PAAA**.

Figure 8 and Figure S2 show the effects of contact time on the evolution of Pb and Cd adsorption onto **CHIT-PAAA**. For both investigated metals, adsorption occurred quickly in the first hour of interaction with the new material. Maximum adsorption efficiency was registered after 45 min of contact time for Pb and 60 min for Cd, after which equilibrium was reached. 

**Figure 9:**
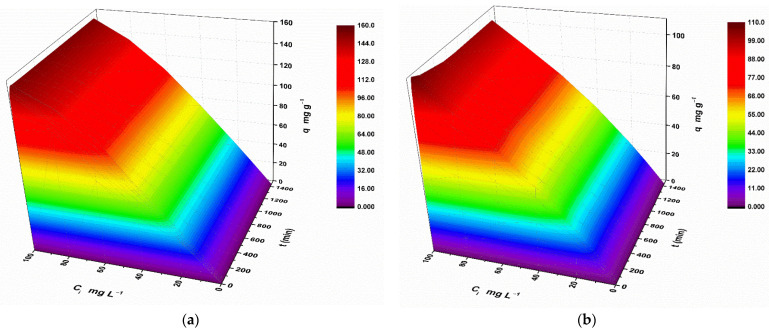
The 3D adsorption models of combined effects of variables on (**a**) Pb and (**b**) Cd adsorption capacity of **CHIT-PAAA**.

Based on the results obtained from the adsorption experiments, 3D adsorption models were developed for each metal. As can be seen in Figure 9, sorption capacity was directly influenced by contact time and initial metal concentrations, highlighting proportional dependencies between these variables.

**Figure 10:**
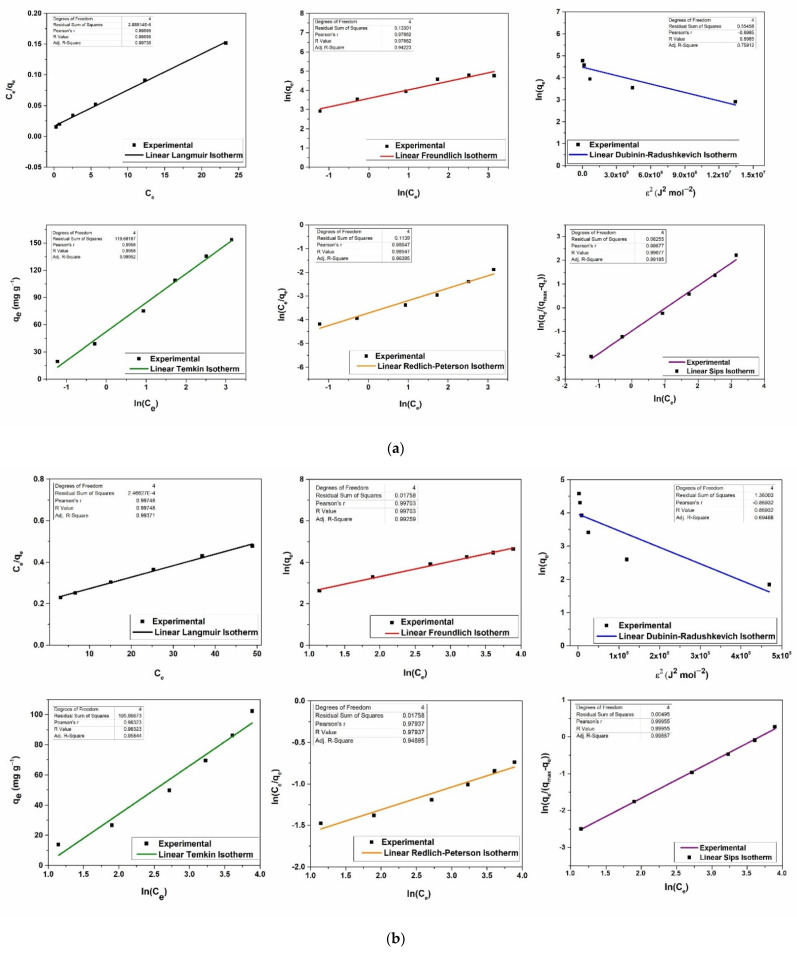
Linear forms of the equilibrium isotherms obtained for (**a**) Pb and (**b**) Cd adsorption onto **CHIT-PAAA**.

The results obtained from the equilibrium study (Figure 10) show that the linear form of the Langmuir isotherm best fitted the (R^2^ = 0.998) Pb experimental sorption data. In contrast, linear Sips (which is a combination of the Langmuir and Freundlich models) was more suitable (R^2^ = 0.999) for Cd. The constants and correlation coefficients resulting from the linear plots of the applied isotherms are summarized in Table S4. The maximum sorption capacities of **CHIT-PAAA** were 170.068 mg g^−1^ for Pb and 180.505 mg g^−1^ for Cd. Pb and Cd adsorption processes could both be categorized as favorable based on the calculated values of the separation factors (R_L_ ranged from 0.00006 to 0.00058) (Figure S3). The Sips model indicated that at low C_e_ values (ẞ_S_ between 0–1), the isotherm reduced to Freundlich characteristics, while at high C_e_ values (ẞ_S_ approaches 1), it highlighted the Langmuir monolayer sorption features [98].

**Figure 11:**
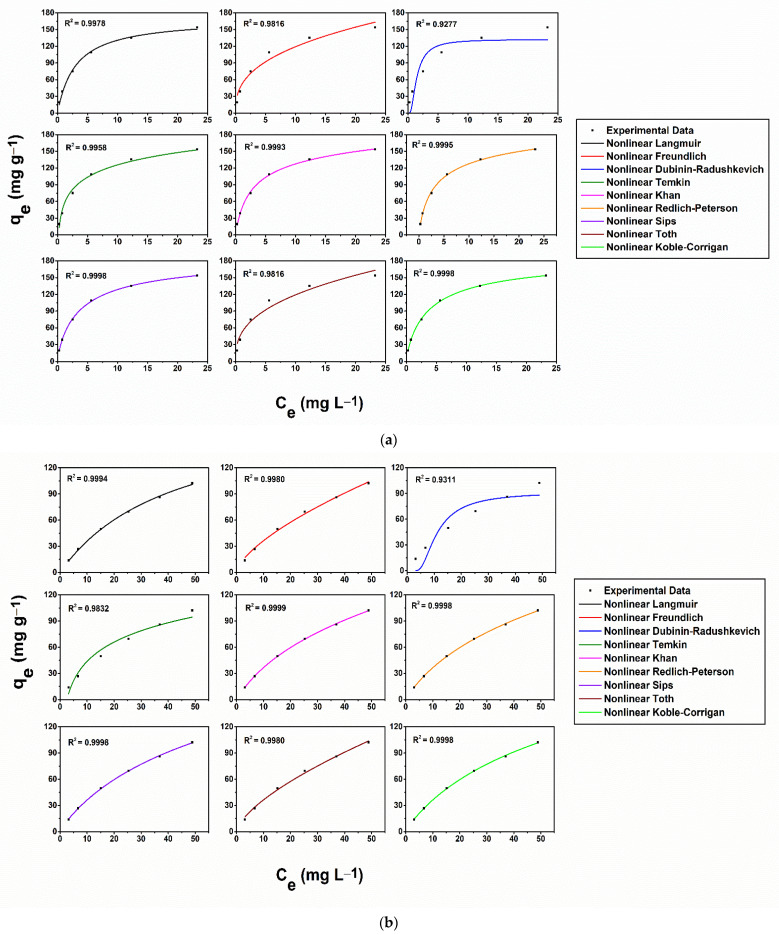
Nonlinear forms of the equilibrium isotherms obtained for (**a**) Pb and (**b**) Cd adsorption onto **CHIT-PAAA**.

The nonlinear fitting of the experimental data is presented in Figure 11. Best fit was recorded with Sips and Koble–Corrigan isotherms (R^2^ = 0.999; RMSE = 1.339) for Pb adsorption data and with the Khan isotherm (R^2^ = 0.999; RMSE = 0.636) for Cd. Pb and Cd isotherm shapes were generally attributed to the class L subgroup 2 type, with and without strict plateaus, respectively, based on Giles et al. [99] and Essington [100] categorizations.

**Figure 12:**
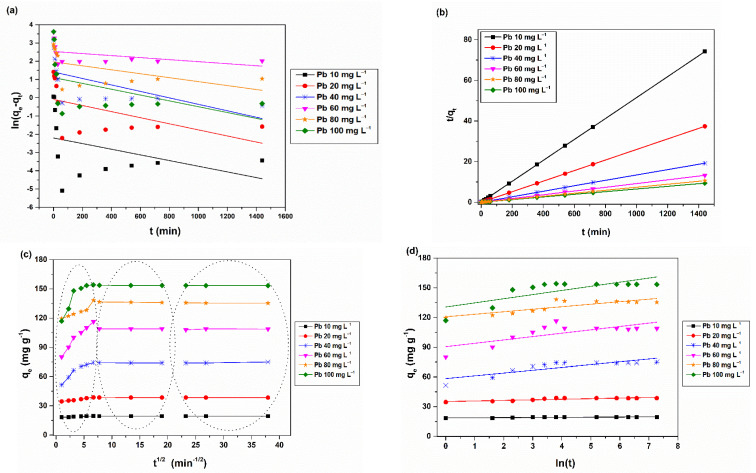
(**a**) Pseudo-first order, (**b**) pseudo-second order, (**c**) Weber–Morris intra-particle diffusion, and (**d**) Elovich kinetic models used to describe Pb adsorption on **CHIT-PAAA**.

**Figure 13:**
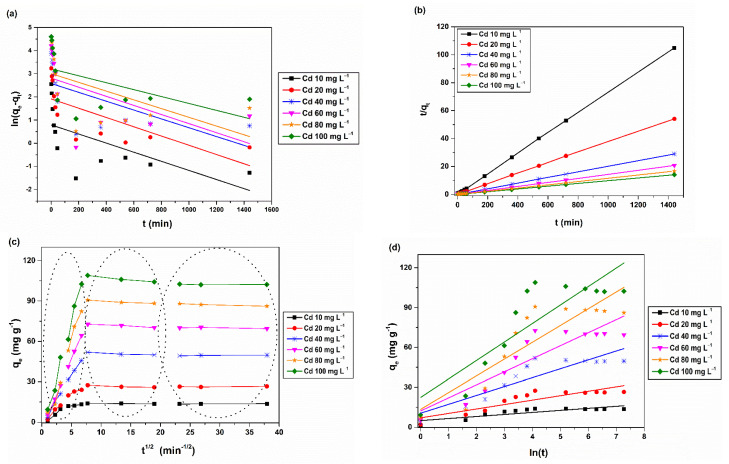
(**a**) Pseudo-first order, (**b**) pseudo-second order, (**c**) Weber–Morris intra-particle diffusion, and (**d**) Elovich kinetic models used to describe Cd adsorption on **CHIT-PAAA**.

Kinetics is one of the most important characteristics of the adsorption process and describes the uptake rate depending on the contact time. Linear regression was performed on the four kinetic models applied to the Pb and Cd adsorption data (Figure 12 and Figure 13). The correlation coefficients (R^2^) and the differences between the calculated (q_e_) and experimental amounts of metals adsorbed (q_e1_, q_e2_) were taken into consideration to determine the kinetic model that best described the sorption process onto **CHIT-PAAA** (Table S5). Comparing the correlation coefficients obtained, the data followed the sequence: Pseudo-second order kinetics (R^2^ varied between 0.999–1) > Elovich kinetics (R^2^ varied between 0.774–0.898) > Weber–Morris intra-particle diffusion (R^2^ varied between 0.450–0.670) > pseudo-first order kinetics (R^2^ varied between 0.387–0.715). Pseudo-second order kinetics provided the most appropriate model for the characterization of both Pb and Cd sorption mechanisms, indicating that chemisorption was the process influencing the rates of adsorption. This fact was also supported by the BET measurements (St < 1 m^2^ g^−1^), highlighting that for **CHIT-PAAA** the mechanism of adsorption relies mainly on the chelation of metal ions by the functional groups present in the structure of the copolymer. Nevertheless, the results obtained for τ12 showed that **CHIT-PAAA** required between 1s–2 min and 4–7 min to reach half the adsorption capacities for Pb and Cd, respectively.

**Figure 14:**
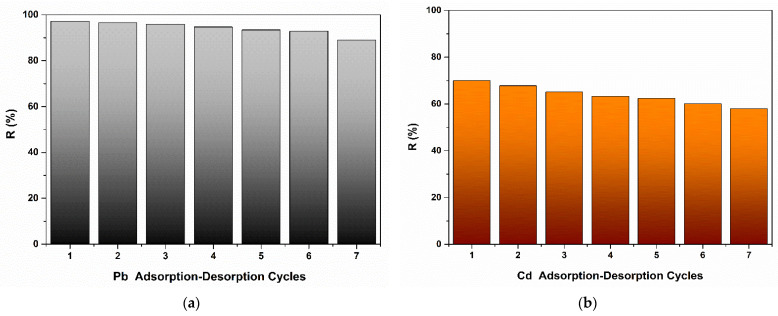
**CHIT-PAAA** recyclability study performed for (**a**) Pb and (**b**) Cd adsorption.

The conducted recyclability study showed that **CHIT-PAAA** removal efficiency decreased from 97.18% to 89% for Pb and from 70% to 58% for Cd after seven cycles of adsorption–desorption (Figure 14).

**Figure 15:**
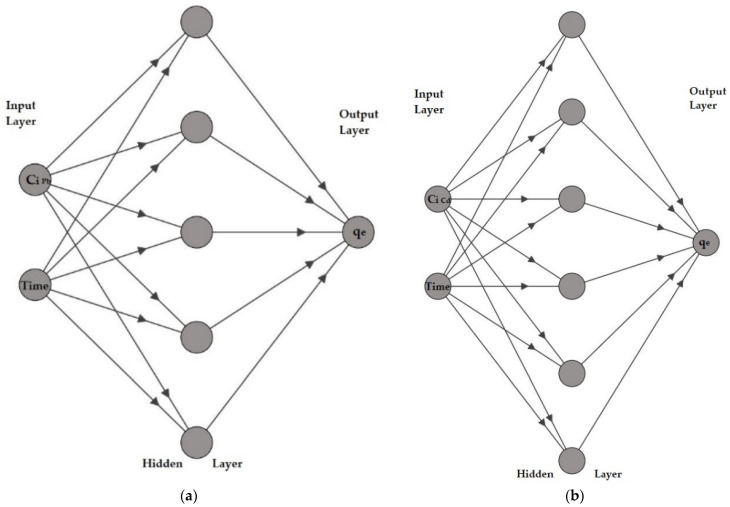
ANN architectures for (**a**) Pb and (**b**) Cd adsorption modeling onto **CHIT-PAAA**.

The ANN architectures developed for models of Pb and Cd adsorption onto **CHIT-PAAA** are presented in Figure 15. Eight algorithms were trained for each ANN: Levenberg–Marquardt backpropagation, resilient backpropagation, Fletcher–Reeves conjugate gradient backpropagation, Polak–Ribiére conjugate gradient backpropagation, Powell–Beale conjugate gradient backpropagation, scaled conjugate gradient backpropagation, BFGS Quasi-Newton gradient backpropagation and one-step secant backpropagation (Table S6). It was determined that the Levenberg–Marquardt design was the most suitable algorithm to model both metals’ adsorption processes (Figure S4). The algorithm selection was achieved by checking the highest R^2^ values and the lowest MSEs for Pb R^2^ = 0.999 and MSE = 8.88 10^−2^, and for Cd R^2^ = 0.999 and MSE = 7.89 10^−2^. The optimum number of hidden neurons was five in the case of Pb and six for Cd sorption. The predicted ANN results were very close to the experimental data, highlighting a good fit and a low MSE (Table S7).

### 3.3. Assays on Metal-Polluted Water Samples

**Figure 16:**
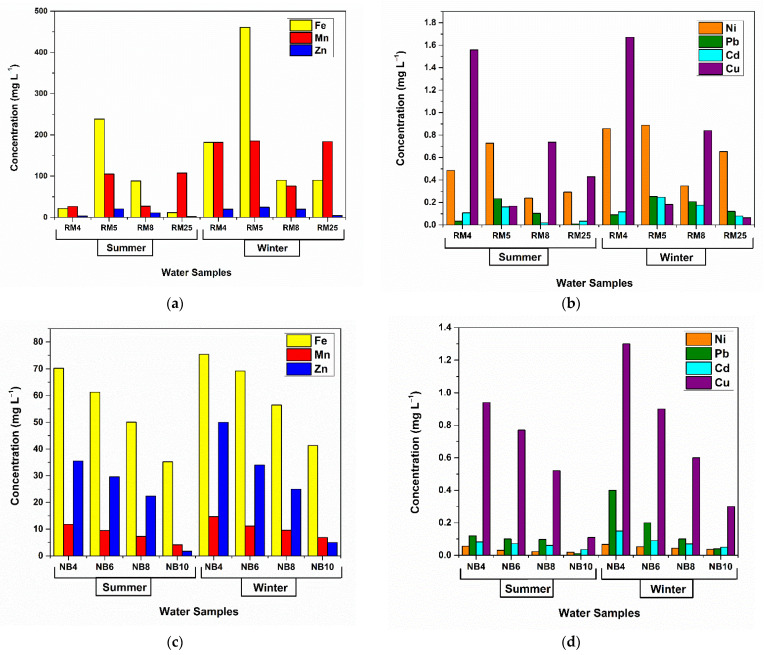
Heavy metals concentrations measured in water samples from (**a**,**b**) Roșia Montană and (**c**,**d**) Novăț-Borșa mining areas, Romania.

Adsorption assays were performed on the collected contaminated water samples after establishing the initial metal composition. As can be seen in Figure 16, Roșia Montană water samples registered significant amounts of Fe (11.48–460.81 mg L^−1^), Mn (26.34–185.22 mg L^−1^), Cu (0.06–1.55 mg L^−1^), and Ni (0.24–0.89 mg L^−1^), while Novăț-Borșa samples were rich in Fe (35.21–70.2 mg L^−1^), Zn (1.77–50 mg L^−1^), Cu (0.11–0.94 mg L^−1^) and Pb (0.01–0.40 mg L^−1^). In general, high concentrations were measured for Fe, Mn, and Zn in all investigated samples. Important metal inputs could be noticed during the winter season due to the increase in water flow from rain and snow. Each mining area had a specific metal composition profile as a result of its local geology and geochemistry, and Roșia Montană water samples recorded higher metal concentrations compared with Novăț-Borșa samples.

**Figure 17:**
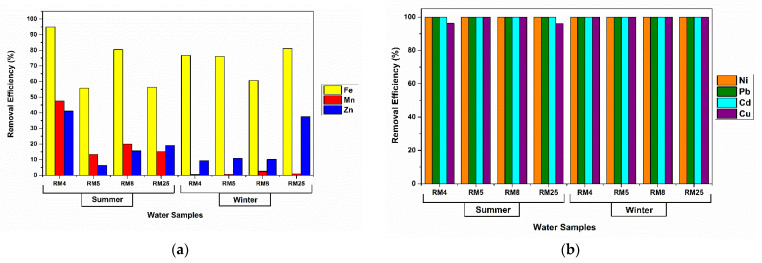

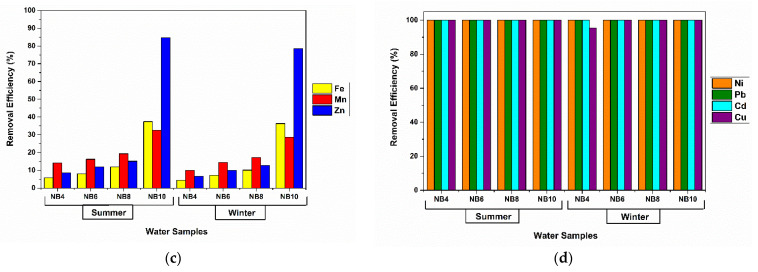
**CHIT-PAAA** removal efficiencies for heavy metals in water samples from (**a**,**b**) Roșia Montană and (**c**,**d**) Novăț-Borșa mining areas, Romania.

On the other hand, excellent removal efficiencies (100%) were obtained for Ni, Pb, Cd, and Cu in water samples collected from both locations (Figure 17). This fact indicates a better adsorption performance of **CHIT-PAAA** at low metal concentrations. Nonetheless, very good adsorption efficiencies were also determined for Fe (up to 95%) in Roșia Montană water samples and Zn (up to 85%) in Novăț-Borșa samples.

### 3.4. XPS Results

**Figure 18:**
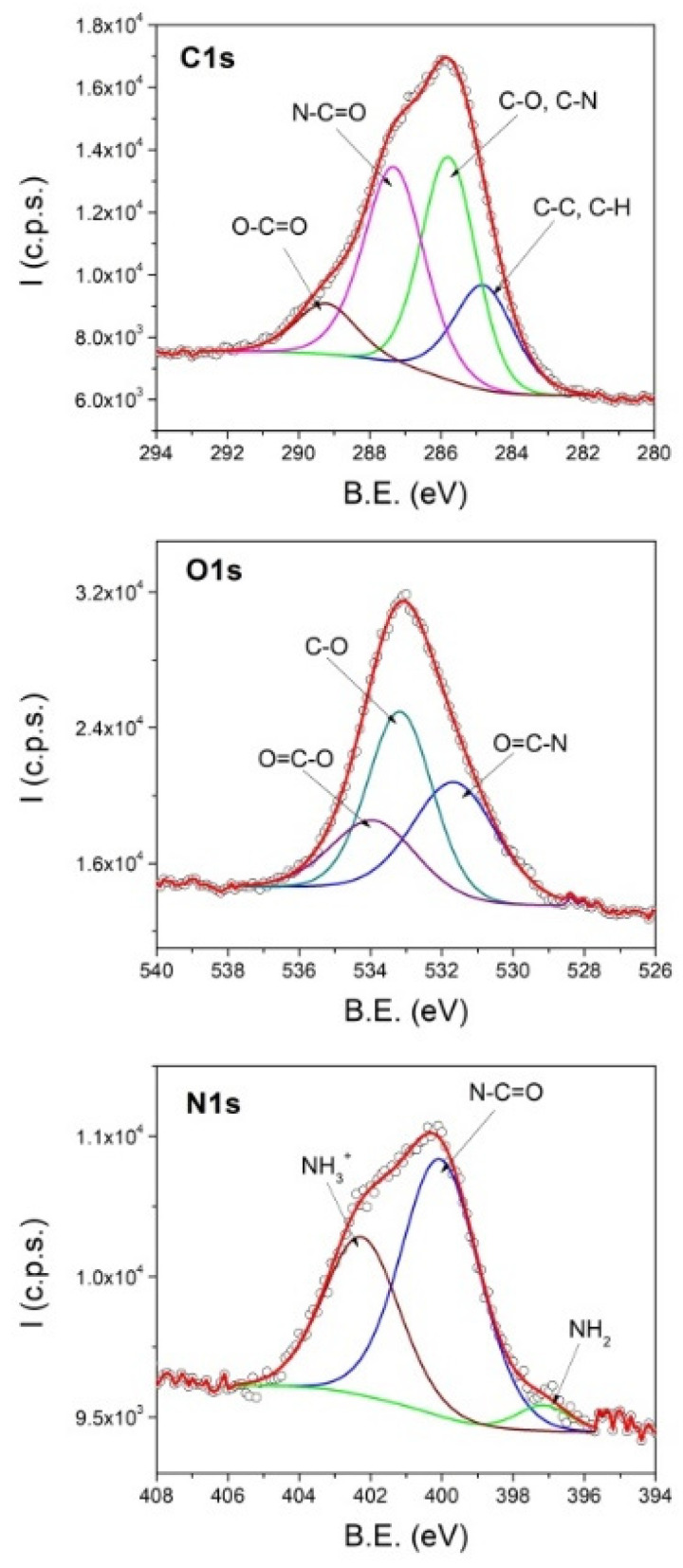
High resolution XPS spectra of C1s, O1s and N1s core levels from **CHIT-PAAA**.

XPS analysis evidences the formation of the copolymer **CHIT-PAAA** and the adsorption of Pb into this copolymer. Figure 18 shows the high-resolution XPS spectra for C1s, O1s, and N1s for the copolymer **CHIT-PAAA**. The best fit for the C1s spectrum was obtained with four components; the component located at 284.8 eV corresponded to C-C, C-H; that at 285.78 eV corresponded to C-N, C-O; the component at 287.6 eV corresponded to the amide group N-C=O which demonstrates the copolymer formation; the higher binding energy component located at 289.2 eV corresponded to the O-C=O group. The N1s spectrum exhibited three components assigned to the nitrogen atoms, from NH_2_, N-C=O groups, and protonated nitrogen NH_3_^+^.

**Figure 19:**
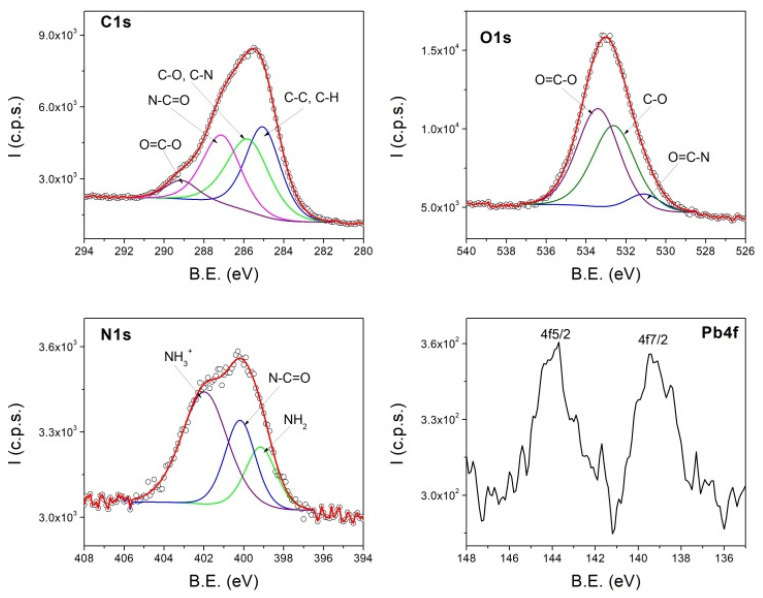
High-resolution XPS spectra of C1s, O1s, N1s and Pb4f core levels from **CHIT-PAAA** after adsorption of Pb^2+^.

The adsorption of Pb on **CHIT-PAAA** is evidenced by the high-resolution XPS spectra shown in Figure 19. The Pb spectrum from Figure 19 exhibits the doublet Pb4f5/2 and Pb 4f7/2 located at 143.7 eV and 138.8 eV, corresponding to Pb. A comparison of the XPS spectra from Figure 18 and Figure 19 shows changes in the relative intensities of the component peaks, especially for O1s and N1s. This fact suggests that the mechanism of Pb adsorption on **CHIT-PAAA** involved the interaction of O and N atoms with metallic ions.

## 4. Conclusions

In conclusion, a new hybrid material was synthesized by green methods through the modification of **CHIT** with **PBAAA**. Successful results were achieved regarding the material’s efficiency and selectivity in retaining Pb (96.07–100%), Cd (76.71–100%), Fe (95%), Zn (85%), Ni (100%), Cu (100%) from batch solutions and contaminated mining water samples. Maximum adsorption was reached quickly, after only 45 min contact time for Pb and 60 min for Cd. The applied 3D, equilibrium, and kinetic models suggested that the sorption capacity of **CHIT-PAAA** was directly dependent on the contact time and initial metal concentrations, and chemisorption was the rate-limiting process. Similar results were generated with the neural network architectures developed, highlighting a high level of trust in the ANN models for both Pb and Cd adsorption. The reciclability study of the copolymer indicated that the removal efficiency decreased to 89% for Pb and 58% for Cd after seven adsorption–desorption cycles. The results of the present investigation suggest that the newly synthesized material is cost-effective, eco-friendly, and has excellent performance in removing metal ions, being suitable for applications in the field of water and wastewater treatment technologies. Therefore, this new copolymer can be used to remediate the issue of contaminated waters, reducing heavy metal pollution, and promoting sustainable development.

## Figures and Tables

**Table 1:** Comparison of Pb and Cd adsorption capacities reported in the literature for different adsorbent materials.

Heavy Metal	q_max_ (mg g^−1^)	Adsorbent Material	Reference
Cd	1.06	Chitosan	[88]
Cd	9.9		[89]
Cd	94		[90]
Pb	7.64		[77]
Pb	34.98		[79]
Pb	55.5		[91]
Pb	34.13	Epichlorohydrin crosslinked chitosan	[78]
Pb	63.33	Chitosan–magnetite	[80]
Pb	112.98	Magnetic chitosan nanocomposites	[81]
Pb	142.67	Geopolymer–alginate– chitosan	[82]
Pb	189.60	Magnetic chitosan functionalized with EDTA	[92]
Pb	334.90	Crosslinked carboxylated chitosan–carboxylated nanocellulose hydrogel beads	[93]
Pb	441.20	Polydopamine-modified chitosan	[94]
Cd	344.00	Chitosan–activated- carbon–iron bio-nanocomposite	[95]
Pb	11.98	Chitosan bead-supported MnFe_2_O_4_ nanoparticles	[83]
Cd	9.73		
Pb	13.23	Polyaniline-grafted chitosan	[84]
Cd	12.87		
Pb	86.09	Chitosan-coated cotton fibers	[85]
Cd	14.14		
Pb	96.62	Chitosan-*g*-methylenebisacrylamide/poly(acrylic acid)	[86]
Cd	80.57		
Pb	125.40	Activated carbon–chitosan complex	[87]
Cd	69.40		
Pb	395.00	Crosslinked glucan–chitosan	[96]
Cd	269.00		
Pb	447.00	Chitosan–sulfhydryl-functionalized graphene oxide composites	[97]
Cd	177.00		
**Pb**	**170.07**	**CHIT-PAAA**	**Current Study**
**Cd**	**180.51**		

## Data Availability

No new data were created or analyzed in this study. Data sharing is not applicable to this article.

## Supplementary Materials

The following are available online at https://www.mdpi.com/article/10.3390/polym14183735/s1, Table S1: Isotherm models used on **CHIT-PAAA** adsorption data, Table S2: Kinetic models used on **CHIT-PAAA** adsorption data, Table S3: Geographic coordinates of the collected metal-polluted water samples, Figure S1: FTIR of **CHIT-PAAA** before and after adsorption of Pb and Cd from 40 mg L^−1^ (**a**) and 100 mg L^−1^ (**b**) stock solutions, Figure S2: The effect of contact time on (**a**) Pb and (**b**) Cd sorption capacities of **CHIT-PAAA,** Table S4. Results of the Pb and Cd adsorption equilibrium study performed on **CHIT-PAAA.** (Ci = 10–100 mg L^−1^, 0.02 g material, 298 K, 600 rpm, 24 h), Figure S3. Separation factors determined for Pb (**a**) and Cd (**b**) adsorption onto **CHIT-PAAA,** Table S5: Results of the kinetic models applied for Pb and Cd sorption onto **CHIT-PAAA** (Ci = 10–100 mg L−1, 0.04 g material, 298 K, 600 rpm), Table S6: Performance of algorithms applied for ANN modeling of Pb and Cd adsorption onto **CHIT-PAAA**, Figure S4: Error histograms and regression plots of Pb (a,b) and Cd (c,d) ANN adsorption models generated with Levenberg–Marquardt algorithm, Table S7: Comparison between calculated and ANN predicted values of the metal amount adsorbed onto r.

## References

[1] Schwarzenbach R.P., Escher B.I., Fenner K., Hofstetter T.B., Johnson C.A., Von Gunten U., Wehrli B. (2006). The Challenge of Micropollutants in Aquatic Systems. Science.

[2] Delpla I., Jung A.V., Baures E., Clement M., Thomas O. (2009). Impacts of Climate Change on Surface Water Quality in Relation to Drinking Water Production. Environ. Int..

[3] Kallenborn R. (2006). Persistent Organic Pollutants (POPs) as Environmental Risk Factors in Remote High-Altitude Ecosystems. Ecotoxicol. Environ. Saf..

[4] Dudgeon D., Arthington A.H., Gessner M.O., Kawabata Z.-I., Knowler D.J., Lévêque C., Naiman R.J., Ne Prieur-Richard A.-H., Soto D., Stiassny M.L.J. (2006). Freshwater Biodiversity: Importance, Threats, Status and Conservation Challenges. Cambridge Philos. Soc..

[5] Schindler D.E., Hilborn R. (2015). Prediction, Precaution, and Policy under Global Change. Emphasize Robustness, Monitoring, and Flexibility. Science.

[6] Malmqvist B., Rundle S. (2002). Threats to the Running Water Ecosystems of the World. Environ. Conserv..

[7] Narayan S.S., Dipak P. (2014). Heavy Metal Tolerance and Accumulation by Bacterial Strains Isolated from Waste Water. J. Chem. Biol. Phys. Sci..

[8] Agency for Toxic Substances and Disease Registry (ATSDR) (2007). Toxicological Profile for Lead.

[9] Agency for Toxic Substances and Disease Registry (ATSDR) (2012). Toxicological Profile for Cadmium.

[10] Wan Ngah W.S., Teong L.C., Hanafiah M.A.K.M. (2011). Adsorption of Dyes and Heavy Metal Ions by Chitosan Composites: A Review. Carbohydr. Polym..

[11] Reddy D.H.K., Lee S.M. (2013). Application of Magnetic Chitosan Composites for the Removal of Toxic Metal and Dyes from Aqueous Solutions. Adv. Colloid Interface Sci..

[12] Varma A.J., Deshpande S.V., Kennedy J.F. (2004). Metal Complexation by Chitosan and Its Derivatives: A Review. Carbohydr. Polym..

[13] Guibal E. (2004). Interactions of Metal Ions with Chitosan-Based Sorbents: A Review. Sep. Purif. Technol..

[14] Fang C., Xiong Z., Qin H., Huang G., Liu J., Ye M., Feng S., Zou H. (2014). One-Pot Synthesis of Magnetic Colloidal Nanocrystal Clusters Coated with Chitosan for Selective Enrichment of Glycopeptides. Anal. Chim. Acta.

[15] Li N., Bai R. (2006). Highly Enhanced Adsorption of Lead Ions on Chitosan Granules Functionalized with Poly(Acrylic Acid). Ind. Eng. Chem. Res..

[16] Charpentier T.V.J., Neville A., Lanigan J.L., Barker R., Smith M.J., Richardson T. (2016). Preparation of Magnetic Carboxymethylchitosan Nanoparticles for Adsorption of Heavy Metal Ions. ACS Omega.

[17] Mahaweero T. (2013). Extraction of Heavy Metals from Aqueous Solutions Using Chitosan/Montmorillonite Hybrid Hydrogels. Master’s Thesis.

[18] Zhang L., Zeng Y., Cheng Z. (2016). Removal of Heavy Metal Ions Using Chitosan and Modified Chitosan: A Review. J. Mol. Liq..

[19] Gokila S., Gomathi T., Sudha P.N., Anil S. (2017). Removal of the Heavy Metal Ion Chromiuim(VI) Using Chitosan and Alginate Nanocomposites. Int. J. Biol. Macromol..

[20] Medeiros Borsagli F.G.L., Mansur A.A.P., Chagas P., Oliveira L.C.A., Mansur H.S. (2015). O-Carboxymethyl Functionalization of Chitosan: Complexation and Adsorption of Cd (II) and Cr (VI) as Heavy Metal Pollutant Ions. React. Funct. Polym..

[21] Sun X., Li Q., Yang L., Liu H. (2016). Chemically Modified Magnetic Chitosan Microspheres for Cr(VI) Removal from Acidic Aqueous Solution. Particuology.

[22] Kulkarni P.S., Deshmukh P.G., Jakhade A.P., Kulkarni S.D., Chikate R.C. (2017). 1,5 Diphenyl Carbazide Immobilized Cross-Linked Chitosan Films: An Integrated Approach towards Enhanced Removal of Cr(VI). J. Mol. Liq..

[23] Gopal Reddi M.R., Gomathi T., Saranya M., Sudha P.N. (2017). Adsorption and Kinetic Studies on the Removal of Chromium and Copper onto Chitosan-g-Maliec Anhydride-g-Ethylene Dimethacrylate. Int. J. Biol. Macromol..

[24] Anitha T., Senthil Kumar P., Sathish Kumar K., Ramkumar B., Ramalingam S. (2015). Adsorptive Removal of Pb(II) Ions from Polluted Water by Newly Synthesized Chitosan–Polyacrylonitrile Blend: Equilibrium, Kinetic, Mechanism and Thermodynamic Approach. Process Saf. Environ. Prot..

[25] Sobahi T.R.A., Abdelaal M.Y., Makki M.S.I. (2014). Chemical Modification of Chitosan for Metal Ion Removal. Arab. J. Chem..

[26] Dubey R., Bajpai J., Bajpai A.K. (2016). Chitosan-Alginate Nanoparticles (CANPs) as Potential Nanosorbent for Removal of Hg (II) Ions. Environ. Nanotechnol. Monit. Manag..

[27] Gupta V.K., Gupta D., Agarwal S., Kothiyal N.C., Asif M., Sood S., Pathania D. (2016). Fabrication of Chitosan-g-Poly(Acrylamide)/Cu Nanocomposite for the Removal of Pb(II) from Aqueous Solutions. J. Mol. Liq..

[28] Miretzky P., Cirelli A.F. (2009). Hg(II) Removal from Water by Chitosan and Chitosan Derivatives: A Review. J. Hazard. Mater..

[29] Mousa N.E., Simonescu C.M., Pătescu R.E., Onose C., Tardei C., Culiţă D.C., Oprea O., Patroi D., Lavric V. (2016). Pb2 + Removal from Aqueous Synthetic Solutions by Calcium Alginate and Chitosan Coated Calcium Alginate. React. Funct. Polym..

[30] Ji G., Bao W., Gao G., An B., Zou H., Gan S. (2012). Removal of Cu (II) from Aqueous Solution Using a Novel Crosslinked Alumina-Chitosan Hybrid Adsorbent. Chin. J. Chem. Eng..

[31] Lalhmunsiama, Lalchhingpuii;, Nautiyal B.P., Tiwari D., Choi S.I., Kong S.H., Lee S.M. (2016). Silane Grafted Chitosan for the Efficient Remediation of Aquatic Environment Contaminated with Arsenic(V). J. Colloid Interface Sci..

[32] Pal P., Pal A. (2017). Surfactant-Modified Chitosan Beads for Cadmium Ion Adsorption. Int. J. Biol. Macromol..

[33] Kyzas G.Z., Kostoglou M. (2015). Swelling–Adsorption Interactions during Mercury and Nickel Ions Removal by Chitosan Derivatives. Sep. Purif. Technol..

[34] Hu C., Zhu P., Cai M., Hu H., Fu Q. (2017). Comparative Adsorption of Pb(II), Cu(II) and Cd(II) on Chitosan Saturated Montmorillonite: Kinetic, Thermodynamic and Equilibrium Studies. Appl. Clay Sci..

[35] Shankar P., Gomathi T., Vijayalakshmi K., Sudha P.N. (2014). Comparative Studies on the Removal of Heavy Metals Ions onto Cross Linked Chitosan-g-Acrylonitrile Copolymer. Int. J. Biol. Macromol..

[36] Sutirman Z.A., Sanagi M.M., Abd Karim K.J., Wan Ibrahim W.A. (2016). Preparation of Methacrylamide-Functionalized Crosslinked Chitosan by Free Radical Polymerization for the Removal of Lead Ions. Carbohydr. Polym..

[37] Liu Q., Yang B., Zhang L., Huang R. (2015). Adsorptive Removal of Cr(VI) from Aqueous Solutions by Cross-Linked Chitosan/Bentonite Composite. Korean J. Chem. Eng..

[38] Seyedmohammadi J., Motavassel M., Maddahi M.H., Nikmanesh S. (2016). Application of Nanochitosan and Chitosan Particles for Adsorption of Zn(II) Ions Pollutant from Aqueous Solution to Protect Environment. Model. Earth Syst. Environ..

[39] Ganea I.-V., Nan A., Neamt I., Baciu C., Serrano A.R. (2021). Neoteric Material Based on Renewable Resources for Metal-Contaminated Waters. Environ. Sci. Proc..

[40] Nan A., Bunge A., Cîrcu M., Petran A., Hǎdade N.D., Filip X. (2017). Poly(Benzofuran-Co-Arylacetic Acid)—A New Type of Highly Functionalized Polymers. Polym. Chem..

[41] Ortega A., Sánchez A., Burillo G. (2021). Binary Graft of Poly(N-Vinylcaprolactam) and Poly(Acrylic Acid) onto Chitosan Hydrogels Using Ionizing Radiation for the Retention and Controlled Release of Therapeutic Compounds. Polymers.

[42] Langmuir I. (1917). The Constitution and Fundamental Properties of Solids and Liquids. Part II.-Liquids. J. Franklin Inst..

[43] Freundlich H. (1907). Über Die Absorption in Lösungen. Z. Phys. Chem. Stöch. Verwand..

[44] Dubinin M.M., Radushkevich L.V. (1947). The Equation of the Characteristic Curve of Activated Charcoal. Proc. Acad. Sci. USSR Phys. Chem. Sect..

[45] Temkin M.J., Pyzhev V. (1940). Kinetics of Ammonia Synthesis on Promoted Iron Catalysts. Acta Physicochim. URSS.

[46] Khan A.R., Ataullah R., Al-Haddad A. (1994). Equilibrium Adsorption Studies of Some Aromatic Pollutants from Dilute Aqueous Solutions on Activated Carbon at Different Temperatures. J. Colloid Interface Sci..

[47] Redlich O., Peterson D.L. (1959). A Useful Adsorption Isotherm. J. Phys. Chem..

[48] Sips R. (1948). Combined Form of Langmuir and Freundlich Equations. J. Phys. Chem..

[49] Toth J. (1971). State Equation of the Solid Gas Interface Layer. Acta Chim..

[50] Koble R.A., Corrigan T.E. (1952). Adsorption Isotherms for Pure Hydrocarbons. Ind. Eng. Chem..

[51] Hamzaoui M., Bestani B., Benderdouche N. (2018). The Use of Linear and Nonlinear Methods for Adsorption Isotherm Optimization of Basic Green 4-Dye onto Sawdust-Based Activated Carbon. J. Mater. Environ. Sci..

[52] Kocadagistan B., Kocadagistan E. (2014). The Effects of Sunflower Seed Shell Modifying Process on Textile Dye Adsorption: Kinetic, Thermodynamic and Equilibrium Study. Desalination Water Treat..

[53] Lagergren S., Sven K. (1898). Zur Theorie Der Sogennanten Adsorptiongeloster Stoffe. K. Sevenska Vetenskapsakademiens. Handl..

[54] Ho Y.S., McKay G. (2000). The Kinetics of Sorption of Divalent Metal Ions onto Sphagnum Moss Peat. Water Res..

[55] Weber W.J., Morris J.C. (1963). Kinetic of Adsorption on Carbon from Solution. Am. Soc. Civ. Eng..

[56] Zeldowitsch J. (1934). Über Den Mechanismus Der Katalytischen Oxidation Von CO a MnO2. URSS Acta Physiochim..

[57] Marczewski A.W. (2010). Application of Mixed Order Rate Equations to Adsorption of Methylene Blue on Mesoporous Carbons. Appl. Surf. Sci..

[58] Derylo-Marczewska A., Marczewski A.W., Winter S., Sternik D. (2010). Studies of Adsorption Equilibria and Kinetics in the Systems: Aqueous Solution of Dyes–Mesoporous Carbons. Appl. Surf. Sci..

[59] Tvrdík J., Křivý I., Mišík L. (2007). Adaptive Population-Based Search: Application to Estimation of Nonlinear Regression Parameters. Comput. Stat. Data Anal..

[60] Roman T., Asavei R.L., Karkalos N.E., Roman C., Virlan C., Cimpoesu N., Istrate B., Zaharia M., Markopoulos A.P., Kordatos K. (2019). Synthesis and Adsorption Properties of Nanocrystalline Ferrites for Kinetic Modeling Development. Int. J. Appl. Ceram. Technol..

[61] Ghaedi M., Ansari A., Nejad P.A., Ghaedi A., Vafaei A., Habibi M.H. (2015). Artificial Neural Network and Bees Algorithm for Removal of Eosin B Using Cobalt Oxide Nanoparticle-Activated Carbon: Isotherm and Kinetics Study. Environ. Prog. Sustain. Energy.

[62] Ghaedi M., Hosaininia R., Ghaedi A.M., Vafaei A., Taghizadeh F. (2014). Adaptive Neuro-Fuzzy Inference System Model for Adsorption of 1,3,4-Thiadiazole-2,5-Dithiol onto Gold Nanoparticales-Activated Carbon. Spectrochim. Acta Part A Mol. Biomol. Spectrosc..

[63] Ghaedi M., Ghaedi A.M., Negintaji E., Ansari A., Vafaei A., Rajabi M. (2014). Random Forest Model for Removal of Bromophenol Blue Using Activated Carbon Obtained from Astragalus Bisulcatus Tree. J. Ind. Eng. Chem..

[64] Elemen S., Akçakoca Kumbasar E.P., Yapar S. (2012). Modeling the Adsorption of Textile Dye on Organoclay Using an Artificial Neural Network. Dye. Pigment..

[65] Despagne F., Massart D.L. (1998). Neural Networks in Multivariate Calibration. Analyst.

[66] Khan T., Mustafa M.R.U., Isa M.H., Manan T.S.B.A., Ho Y.C., Lim J.W., Yusof N.Z. (2017). Artificial Neural Network (ANN) for Modelling Adsorption of Lead (Pb (II)) from Aqueous Solution. Water. Air. Soil Pollut..

[67] Narayana P.L., Maurya A.K., Wang X.S., Harsha M.R., Srikanth O., Alnuaim A.A., Hatamleh W.A., Hatamleh A.A., Cho K.K., Paturi U.M.R. (2021). Artificial Neural Networks Modeling for Lead Removal from Aqueous Solutions Using Iron Oxide Nanocomposites from Bio-Waste Mass. Environ. Res..

[68] Olanrewaju R.F., Mariam R., Ahmed A.A. Modeling of ANN to Determine Optimum Adsorption Capacity for Removal of Pollutants in Wastewater. Proceedings of the 2017 IEEE 4th International Conference on Smart Instrumentation, Measurement and Application (ICSIMA).

[69] McCulloch W.S., Pitts W. (1943). A Logical Calculus of the Ideas Immanent in Nervous Activity. Bull. Math. Biophys..

[70] Mohanraj M., Jayaraj S., Muraleedharan C. (2015). Applications of Artificial Neural Networks for Thermal Analysis of Heat Exchangers—A Review. Int. J. Therm. Sci..

[71] Sha W., Edwards K.L. (2007). The Use of Artificial Neural Networks in Materials Science Based Research. Mater. Des..

[72] Mjalli F.S., Al-Asheh S., Alfadala H.E. (2007). Use of Artificial Neural Network Black-Box Modeling for the Prediction of Wastewater Treatment Plants Performance. J. Environ. Manag..

[73] Cavas L., Karabay Z., Alyuruk H., Doĝan H., Demir G.K. (2011). Thomas and Artificial Neural Network Models for the Fixed-Bed Adsorption of Methylene Blue by a Beach Waste *Posidonia oceanica* (L.) Dead Leaves. Chem. Eng. J..

[74] Chowdhury S., Saha P. (2013). Das Artificial Neural Network (ANN) Modeling of Adsorption of Methylene Blue by NaOH-Modified Rice Husk in a Fixed-Bed Column System. Environ. Sci. Pollut. Res. Int..

[75] Ghaedi A.M., Vafaei A. (2017). Applications of Artificial Neural Networks for Adsorption Removal of Dyes from Aqueous Solution: A Review. Adv. Colloid Interface Sci..

[76] Yildiz S. (2017). Artificial Neural Network (ANN) Approach for Modeling Zn(II) Adsorption in Batch Process. Korean J. Chem. Eng..

[77] Luu T.T., Dinh V.P., Nguyen Q.H., Tran N.Q., Nguyen D.K., Ho T.H., Nguyen V.D., Tran D.X., Kiet H.A.T. (2021). Pb(II) Adsorption Mechanism and Capability from Aqueous Solution Using Red Mud Modified by Chitosan. Chemosphere.

[78] Chen A.H., Liu S.C., Chen C.Y., Chen C.Y. (2008). Comparative Adsorption of Cu(II), Zn(II), and Pb(II) Ions in Aqueous Solution on the Crosslinked Chitosan with Epichlorohydrin. J. Hazard. Mater..

[79] Ngah W.S.W., Fatinathan S. (2010). Pb(II) Biosorption Using Chitosan and Chitosan Derivatives Beads: Equilibrium, Ion Exchange and Mechanism Studies. J. Environ. Sci..

[80] Tran H.V., Tran L.D., Nguyen T.N. (2010). Preparation of Chitosan/Magnetite Composite Beads and Their Application for Removal of Pb(II) and Ni(II) from Aqueous Solution. Mater. Sci. Eng. C.

[81] Rasoulzadeh H., Dehghani M.H., Mohammadi A.S., Karri R.R., Nabizadeh R., Nazmara S., Kim K.H., Sahu J.N. (2020). Parametric Modelling of Pb(II) Adsorption onto Chitosan-Coated Fe3O4 Particles through RSM and DE Hybrid Evolutionary Optimization Framework. J. Mol. Liq..

[82] ALSamman M.T., Sánchez J. (2021). Recent Advances on Hydrogels Based on Chitosan and Alginate for the Adsorption of Dyes and Metal Ions from Water. Arab. J. Chem..

[83] Li H., Ji H., Cui X., Che X., Zhang Q., Zhong J., Jin R., Wang L., Luo Y. (2021). Kinetics, Thermodynamics, and Equilibrium of As(III), Cd(II), Cu(II) and Pb(II) Adsorption Using Porous Chitosan Bead-Supported MnFe_2_O_4_ Nanoparticles. Int. J. Min. Sci. Technol..

[84] Karthik R., Meenakshi S. (2015). Removal of Pb(II) and Cd(II) Ions from Aqueous Solution Using Polyaniline Grafted Chitosan. Chem. Eng. J..

[85] Zhang G., Qu R., Sun C., Ji C., Chen H., Wang C., Niu Y. (2008). Adsorption for Metal Ions of Chitosan Coated Cotton Fiber. J. Appl. Polym. Sci..

[86] Paulino A.T., Belfiore L.A., Kubota L.T., Muniz E.C., Almeida V.C., Tambourgi E.B. (2011). Effect of Magnetite on the Adsorption Behavior of Pb(II), Cd(II), and Cu(II) in Chitosan-Based Hydrogels. Desalination.

[87] Ge H., Fan X. (2011). Adsorption of Pb2+ and Cd2+ onto a Novel Activated Carbon-Chitosan Complex. Chem. Eng. Technol..

[88] Babakhani A., Sartaj M. (2021). Competitive Adsorption of Nickel(II) and Cadmium(II) Ions by Chitosan Cross-Linked with Sodium Tripolyphosphate. Chem. Eng. Commun..

[89] Bassi R., Prasher S.O., Simpson B.K. (2000). Removal of Selected Metal Ions from Aqueous Solutions Using Chitosan Flakes. Sep. Sci. Technol..

[90] Zielińska K., Chostenko A., Truszkowski S. (2010). Adsorption of Cadmium Ions on Chitosan Membranes: Kinetics and Equilibrium Studies. Prog. Chem. Appl. Chitin Deriv..

[91] Sobhanardakani S., Zandipak R., Parvizimosaed H., Khoei A.J., Moslemi M., Tahergorabi M., Hosseini S.M., Delfieh P. (2014). Efficiency of Chitosan for the Removal of Pb (II), Fe (II) and Cu (II) Ions from Aqueous Solutions. Iran. J. Toxicol..

[92] Chen B., Zhao H., Chen S., Long F., Huang B., Yang B., Pan X. (2019). A Magnetically Recyclable Chitosan Composite Adsorbent Functionalized with EDTA for Simultaneous Capture of Anionic Dye and Heavy Metals in Complex Wastewater. Chem. Eng. J..

[93] Xu X., Ouyang X.-K., Yang L.-Y. (2021). Adsorption of Pb(II) from Aqueous Solutions Using Crosslinked Carboxylated Chitosan/Carboxylated Nanocellulose Hydrogel Beads. J. Mol. Liq..

[94] Guo D.-M., An Q.-D., Xiao Z.-Y., Zhai S.-R., Yang D.-J. (2018). Efficient Removal of Pb(II), Cr(VI) and Organic Dyes by Polydopamine Modified Chitosan Aerogels. Carbohydr. Polym..

[95] Sharififard H., Shahraki Z.H., Rezvanpanah E., Rad S.H. (2018). A Novel Natural Chitosan/Activated Carbon/Iron Bio-Nanocomposite: Sonochemical Synthesis, Characterization, and Application for Cadmium Removal in Batch and Continuous Adsorption Process. Bioresour. Technol..

[96] Jiang C., Wang X., Wang G., Hao C., Li X., Li T. (2019). Adsorption Performance of a Polysaccharide Composite Hydrogel Based on Crosslinked Glucan/Chitosan for Heavy Metal Ions. Compos. Part B Eng..

[97] Li X., Zhou H., Wu W., Wei S., Xu Y., Kuang Y. (2015). Studies of Heavy Metal Ion Adsorption on Chitosan/Sulfydryl-Functionalized Graphene Oxide Composites. J. Colloid Interface Sci..

[98] Kumara N.T.R.N., Hamdan N., Petra M.I., Tennakoon K.U., Ekanayake P. (2014). Equilibrium Isotherm Studies of Adsorption of Pigments Extracted from Kuduk-Kuduk (*Melastoma malabathricum* L.) Pulp onto TiO_2_ Nanoparticles. J. Chem..

[99] Giles C.H., Smith D., Huitson A. (1974). A General Treatment and Classification of the Solute Adsorption Isotherm. J. Colloid Interface Sci..

[100] Essington M.E. (2004). Soil and Water Chemistry: An Integrative Approach.

