# Supplementary material for: Development of a New Eco-Friendly Copolymer Based on Chitosan for Enhanced Removal of Pb and Cd from Water
Source: Polymers (Basel). 2022 Sep 7;14(18):3735. doi: 10.3390/polym14183735 (PMC9504173; doi:10.3390/polym14183735)
Supplement: Supplementary file 1 [file polymers-14-03735-s001.zip › polymers-1869699-supplementary.pdf]

polymers

MDPI

Supplementary Materials

Development of a New Eco-Friendly Copolymer Based on Chitosan for Enhanced Removal of Pb and Cd from Water

Iolanda-Veronica Ganea [1,2], Alexandrina Nan [2,*], Carmen Roba [1], Iulia Neamțiu [1,3], Eugen Gurzău [3,4], Rodica Turcu [2], Xenia Filip [2] and Călin Baciu [1,*]

Table S1. Isotherm models used on **CHIT-PAAA** adsorption data

Isotherm Models	Nonlinear Forms	Linear Forms	References
Langmuir	$q_e = \frac{q_{max} \cdot K_L \cdot C_e}{1 + K_L \cdot C_e}$	$\frac{C_e}{q_e} = \frac{C_e}{q_{max}} + \frac{1}{K_L \cdot q_{max}}$	[1]
Freundlich	$q_e = K_F \cdot C_e^{1/n}$	$\ln q_e = \ln K_F + \frac{1}{n} \cdot \ln C_e$	[2]
Dubinin-Radushkevich	$q_e = q_s \cdot \exp(-K_{DR} \cdot \varepsilon^2)$	$\ln q_e = \ln q_s - K_{DR} \cdot \varepsilon^2$	[3]
Temkin	$q_e = \frac{R \cdot T}{K_T} \cdot \ln A_T \cdot C_e$	$q_e = \frac{R \cdot T}{K_T} \cdot \ln A_T + \frac{R \cdot T}{K_T} \cdot \ln C_e$	[4]
Redlich-Peterson	$q_e = \frac{K_R \cdot C_e}{1 + \alpha_R \cdot C_e^g}$	$\ln(\frac{C_e}{q_e}) = \alpha_R \cdot \ln C_e + \ln K_R$	[5]
Sips	$q_e = \frac{q_{max} \cdot K_S \cdot C_e^{\beta_S}}{1 + K_S \cdot C_e^{\beta_S}}$	$\ln\left(\frac{q_e}{q_{max} - q_e}\right) = \beta_S \cdot \ln C_e + \ln(K_S{}^{\beta_S})$	[6]
Toth	$q_e = \frac{K_t \cdot C_e}{(\alpha_t + C_e)^{1/t}}$	-	[7]
Koble-Corrigan	$q_e = \frac{A \cdot C_e^n}{1 + B \cdot C_e^n}$	-	[8]
Khan	$q_e = \frac{q_S \cdot b_K \cdot C_e}{(1 + b_K \cdot C_e)^{\alpha_K}}$	-	[9]

where: q_e is the experimental amount of pollutant adsorbed per unit mass of material (mg g^{-1}); C_e is the concentration of pollutant at the equilibrium state (mg L^{-1}); q_{max} represents the maximum amount of adsorbate (mg g^{-1}); K_L is the constant in the Langmuir adsorption model (L mg^{-1}); K_F (mg ·g^{-1}) and n are both constants in the Freundlich adsorption model; q_s is the theoretical isotherm capacity (mg g^{-1}); ε and K_{DR} are the Dubinin–Radushkevich isotherm constants (mol^2 kJ^{-2}); K_T and A_T are the Temkin isotherm constant and equilibrium binding constant (L g^{-1}); R is the gas constant (J mol^{-1}·K^{-1}); T is the absolute temperature (K); K_R (L g^{-1}) and α_R are the Redlich–Peterson isotherm constants (mg^{-1}); g is the Redlich–Peterson isotherm exponent; K_s is the Sips isotherm model constant (L mg^{-1}) and $ß_S$ is the Sips isotherm model exponent; K_t (mg g^{-1}); α_t (L mg^{-1}) and t are the Toth isotherm constants; α_K and b_K are the Khan isotherm model exponent and constant; A is Koble-Corrigan isotherm constant (L^n·mg^{1-n} ·g^{-1}) and B is Koble-Corrigan isotherm constant ((L mg^{-1})^n).

Table S2. Kinetic models used on **CHIT-PAAA** adsorption data.

Kinetic Model	Linear Form	References
Pseudo-first-order	$\log(q_{e_1} - q_{t_1}) = \log q_{e_1} - \left(\frac{k_1}{2.303}\right) t$	[10]
Pseudo-second-order	$\frac{t}{q_{t_2}} = \frac{1}{k_2 \, q_{e_2}{}^2} + \frac{t}{q_{e_2}}$	[11]
Weber Morris intra-particle diffusion	$q_t = k_{ipd} \cdot t^{1/2} + C$	[12]
Elovich	$q_t = \frac{\ln \alpha \cdot \beta}{\beta} + \frac{1}{\beta} \cdot \ln t$	[13]

where: q_{e_1}, q_{e_2} represent the experimental amount of pollutant adsorbed at the equilibrium state (mg g^{-1}); q_t, q_{t_1}, q_{t_2} (mg g^{-1}) are the amount of pollutant adsorbed at time *t* [min]; k_1 (min^{-1}), k_2 (g g^{-1}·min^{-1}) are the adsorption rate constants of the pseudo-first-order and pseudo-second-order adsorption models; α is the initial adsorption rate (mg g^{-1}·min^{-1}); ß is a

desorption constant related to the extent of surface coverage and activation energy for chemisorption (g mg^{-1}); k_{ipd} is the intra-particle diffusion rate constant (mg g^{-1}·min^{-1}); C is the intercept that gives an idea about the thickness of the boundary layer (mg g^{-1}). K_1 and K_2 parameters and q_e were calculated from the intercepts and slopes obtained by plotting ln(q_e-q_t) versus t and t/q_t versus t, respectively. α and ß Elovich maximum adsorption capacity and constant were calculated from the slopes and the intercepts obtained by plotting q_t versus ln(()t), while K_{ipd} was determined from the plot of q_t versus $t^{1/2}$.

Table S3. Geographic coordinates of the collected metal-polluted water samples.

Sample	Mining Site	Coordinates	
		Latitude	Longitude
RM4	Roșia Montană	46°18′16′′N	23°06′09′′E
RM6	Roșia Montană	46°18′23′′N	23°05′00′′E
RM8	Roșia Montană	46°18′04′′N	23°07′06′′E
RM25	Roșia Montană	46°17′31′′N	23°07′22′′E
NB4	Novăț-Borșa	47°43′10′′N	24°34′41′′E
NB6	Novăț-Borșa	47°43′19′′N	24°34′29′′E
NB8	Novăț-Borșa	47°43′19′′N	24°34′17′′E
NB10	Novăț-Borșa	47°43′29′′N	24°34′21′′E

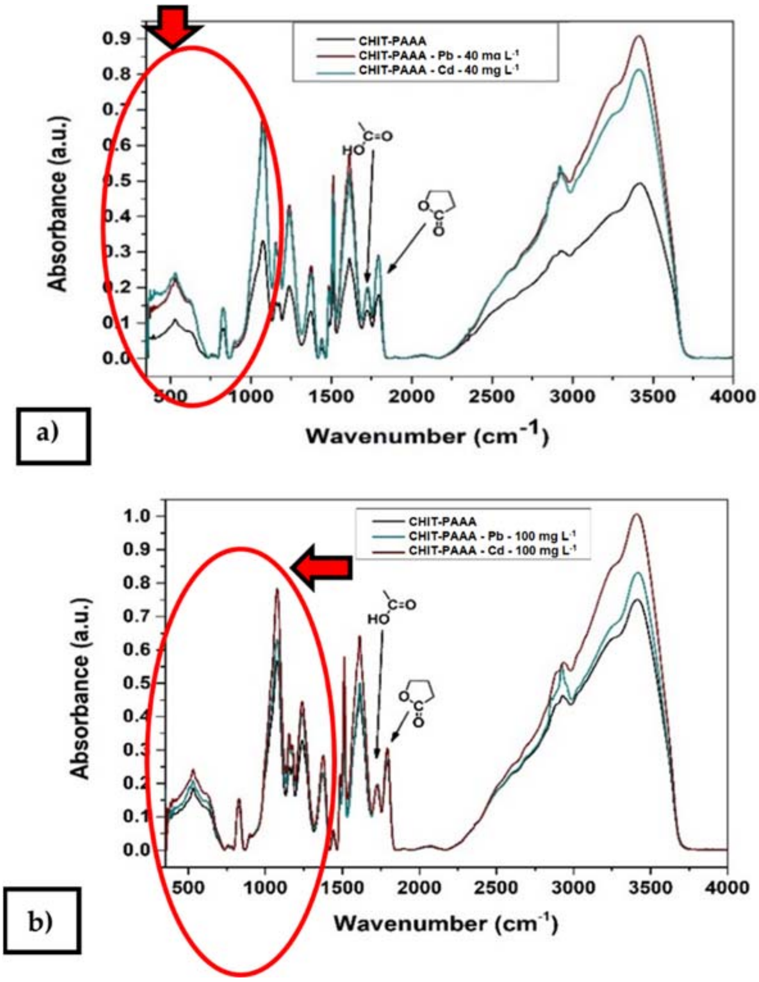

Figure S1. FTIR of **CHIT-PAAA** before and after adsorption of Pb and Cd from 40 mg L^{-1} **(a)** and 100 mg L^{-1} **(b)** stock solutions.

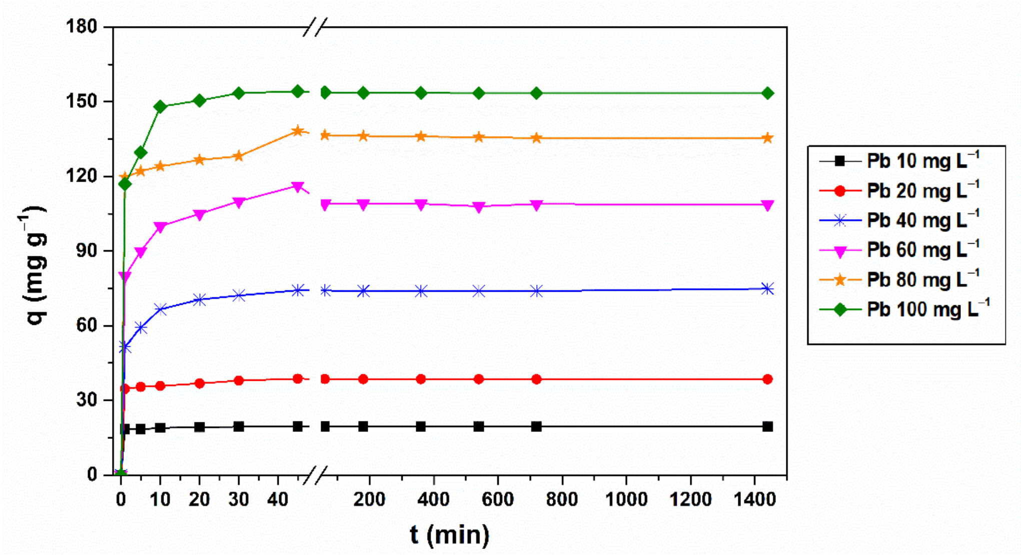

(a)

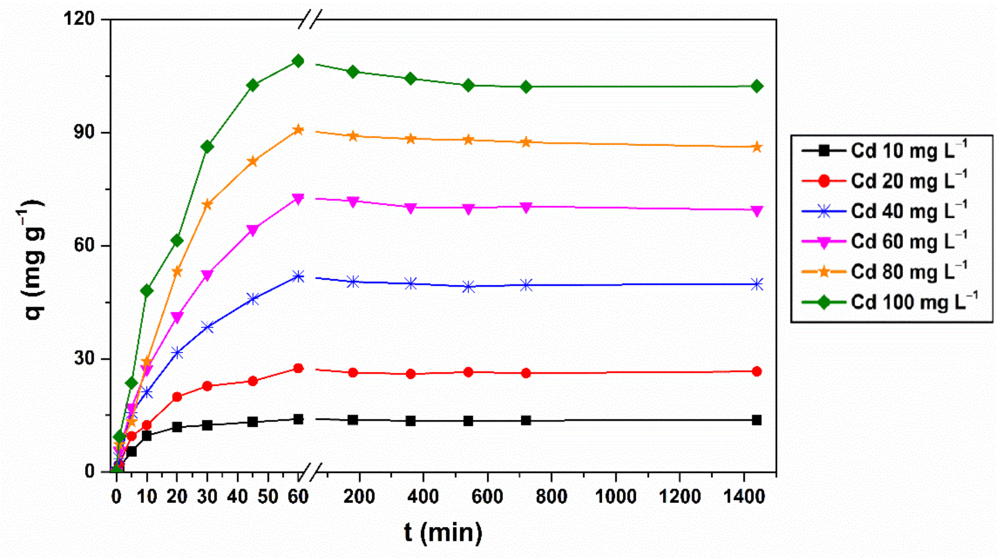

(b)

Figure S2. The effect of contact time on Pb **(a)** and Cd **(b)** sorption capacities of **CHIT-PAAA**.

Table S4. Results of the Pb and Cd adsorption equilibrium study performed on **CHIT-PAAA.** (Ci = 10–100 mg L^{-1}, 0.02 g material, 298 K, 600 rpm, 24 h).

Isotherms	Forms	Coefficients	Pb	Cd
**Langmuir**	Linear	K_L (L mg^{-1})	0.350	0.030
		q_{max} (mg g^{-1})	170.068	180.505
		R^2	0.999	0.998
		RMSE	0.003	0.007
	Nonlinear	K_L (L mg^{-1})	0.330	0.023
		q_{max} (mg g^{-1})	170.343	188.721
		R^2	0.997	0.999
		RMSE	3.903	1.302
**Freundlich**	Linear	K_F (mg g^{-1})	35.909	6.391
		n	2.252	1.375

		R^2	0.977	0.997
		RMSE	0.182	0.066
	Nonlinear	K_F (mg g^{-1})	50.460	7.914
		n	2.681	1.511
		R^2	0.981	0.998
		RMSE	11.385	2.414
**Dubinin-Radushkevich**	Linear	K_{DR} (mol^2 kJ^{-2})	0.001	0.005
		q_s (mg g^{-1})	88.677	52.668
		E (kJ mol^{-1})	19.780	10.011
		R^2	0.899	0.869
		RMSE	0.372	0.583
	Nonlinear	K_{DR} (mol^2 kJ^{-2})	4.618 10^{-7}	16.540 10^{-6}
		q_s (mg g^{-1})	132.555	91.859
		E (kJ mol^{-1})	1.040	0.175
		R^2	0.927	0.931
		RMSE	22.305	13.994
**Temkin**	Linear	A_T (L g^{-1})	1.843	2.549
		K_T	47.476	77.448
		R^2	0.996	0.983
		RMSE	5.469	6.999
	Nonlinear	A_T (L g^{-1})	5.133	0.392
		K_T	77.657	77.446
		R^2	0.995	0.983
		RMSE	5.469	6.999
**Redlich-Peterson**	Linear	K_R (L g^{-1})	41.305	6.391
		α_R (mg^{-1})	0.525	0.272
		R^2	0.986	0.979
		RMSE	0.168	0.066
	Nonlinear	K_R (L g^{-1})	74.333	5.432
		α_R (mg^{-1})	0.624	0.087
		g	0.888	0.748
		R^2	0.999	0.999
		RMSE	2.151	0.738
**Sips**	Linear	q_{max} (L mg^{-1})	170.068	180.505
		K_s (L mg^{-1})	1.00008	0.026
		$ß_S$	0.005	0.992
		R^2	0.997	0.999
		RMSE	0.002	0.035
	Nonlinear	q_{max} (L mg^{-1})	59.438	5.487
		K_s (L mg^{-1})	0.312	0.022
		$ß_S$	0.824	0.889
		R^2	0.999	0.999
		RMSE	1.339	0.916
**Toth**	Nonlinear	K_t	50.474	7.917
		α_t	1.074 10^{-16}	1.430 10^{-17}
		t	1.594	2.955
		R^2	0.981	0.998
		RMSE	13.147	2.787
**Koble-Corrigan**	Nonlinear	A	59.438	5.487
		n	0.824	0.889
		B	0.312	0.022

		R^2	0.999	0.999
		RMSE	1.339	0.916
**Khan**	Nonlinear	q_S	111.567	66.047
		b_K	0.619	0.076
		α_K	0.857	0.569
		R^2	0.999	0.999
		RMSE	2.459	0.636

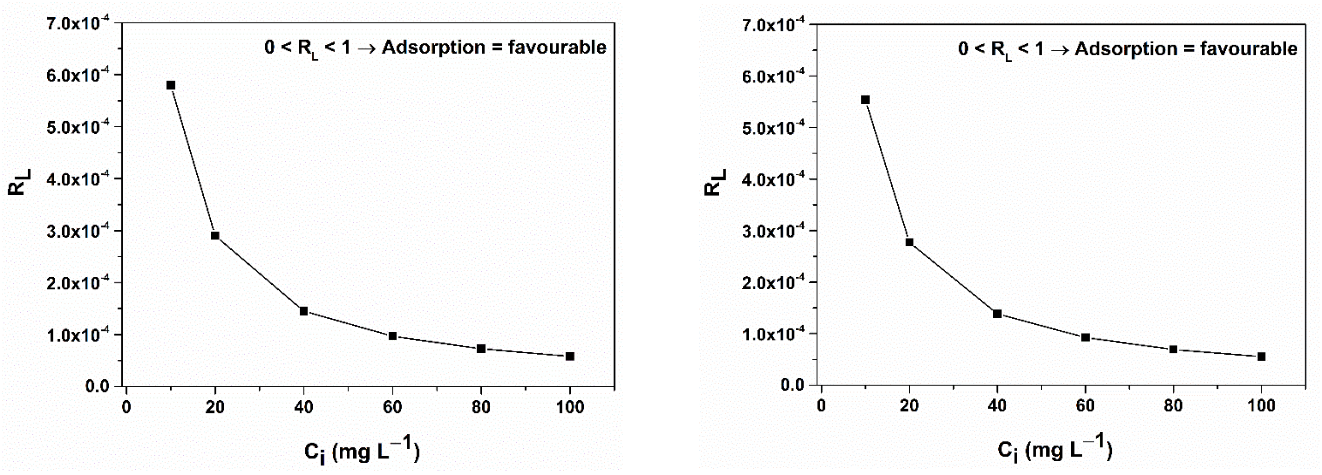

(a) **(b)**

Figure S3. Separation factors determined for Pb **(a)** and Cd **(b)** adsorption onto **CHIT-PAAA.**

Table S5. Results of the kinetic models applied for Pb and Cd sorption onto **CHIT-PAAA** (Ci = 10–100 mg L^{-1}, 0.04 g material, 298 K, 600 rpm).

C_i (mg L^{-1})	q_e (mg g^{-1})	Pseudo-First Order			Pseudo-Second Order				Weber Morris Intra-Particle Diffusion			Elovich		
		k_1 (min^{-1})	q_{e1} (mg g^{-1})	R^2	$\tau_{1/2}$ (min)	k_2 (g mg^{-1} min^{-1})	q_{e2} (mg g^{-1})	R^2	k_{ipd} (mg g^{-1} $min^{-1/2}$)	I	R^2	α (mg $g^{-1}\cdot min^{-1}$)	β (mg $g^{-1}\cdot min^{-1}$)	R^2
**Pb**														
**10**	19.410	$2.19\ 10^{-3}$	0.111	0.387	0.027	1.895	19.414	1	0.009	18.957	0.545	$4.60\ 10^{53}$	6.752	0.809
**20**	38.500	$0.08\ 10^{-3}$	0.915	0.526	0.219	0.119	38.506	1	0.031	36.531	0.625	$6.66\ 10^{25}$	1.708	0.866
**40**	74.920	$1.41\ 10^{-3}$	4.105	0.607	1.880	0.007	74.738	0.999	0.163	65.388	0.574	$2.63\ 10^{9}$	0.354	0.845
**60**	108.757	$2.53\ 10^{-3}$	12.600	0.421	0.035	0.265	108.696	0.999	0.242	99.622	0.450	$1.21\ 10^{12}$	0.294	0.746
**80**	135.360	$1.96\ 10^{-3}$	7.145	0.501	0.069	0.106	135.501	1	0.139	126.688	0.630	$1.05\ 10^{21}$	0.393	0.847
**100**	153.422	$1.10\ 10^{-3}$	3.032	0.448	0.169	0.039	153.374	1	0.281	141.611	0.484	$1.57\ 10^{14}$	0.239	0.774
**Cd**														
**10**	13.720	$4.54\ 10^{-3}$	2.207	0.633	4.468	$1.63\ 10^{-2}$	13.736	0.999	0.194	8.867	0.562	39.299	0.651	0.840
**20**	26.620	$4.61\ 10^{-3}$	6.768	0.715	7.132	$5.25\ 10^{-3}$	26.695	0.999	0.450	15.113	0.633	27.690	0.300	0.884
**40**	49.760	$4.40\ 10^{-3}$	13.182	0.645	7.278	$2.74\ 10^{-3}$	50.075	0.999	0.925	26.409	0.660	32.495	0.150	0.898

**60**	69.440	4.51 10^{-3}	16.639	0.572	6.810	2.09 10^{-3}	70.225	0.999	1.378	35.324	0.670	34.233	0.102	0.898
**80**	86.120	4.35 10^{-3}	20.204	0.562	7.280	1.57 10^{-3}	87.336	0.999	1.753	43.359	0.648	36.615	0.079	0.879
**100**	102.260	3.43 10^{-3}	24.805	0.518	5.326	1.82 10^{-3}	102.987	0.999.	1.882	56.037	0.624	69.705	0.072	0.870

Table S6. Performance of algorithms applied for ANN modeling of Pb and Cd adsorption onto **CHIT-PAAA.**

**Algorithm**	**Function**	**Pb**			**Cd**		
		**No. of Neurons**	**MSE**	**R^2**	**No. of Neurons**	**MSE**	**R^2**
Levenberg–Marquardt	*trainlm*	5	8.88 10^{-2}	0.999	6	7.89 10^{-2}	0.999
Resilient Backpropagation	*trainrp*	7	9.37	0.997	15	40.4	0.975
Fletcher–Reeves Conjugate Gradient	*traincgf*	10	5.39	0.998	6	11.4	0.992
Polak–Ribiére Conjugate Gradient	*traincgp*	13	6.04	0.998	7	14.4	0.991
Powell–Beale Conjugate Gradient	*traincgb*	17	4.34	0.998	7	14.2	0.992
Scaled Conjugate Gradient	*trainscg*	15	3.62	0.999	7	13.3	0.990
BFGS Quasi-Newton	*trainbfg*	15	25.90	0.993	23	97.3	0.943
One Step Secant	*trainoss*	18	7.36	0.998	8	20.7	0.988

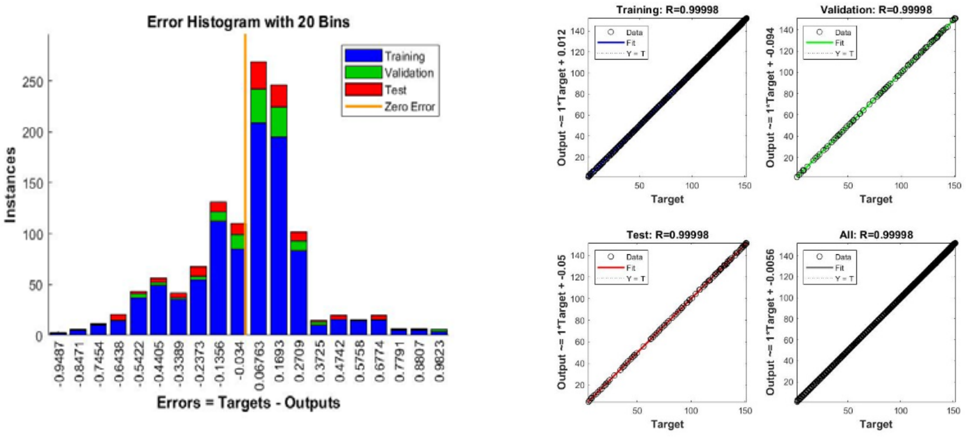

(a) **(b)**

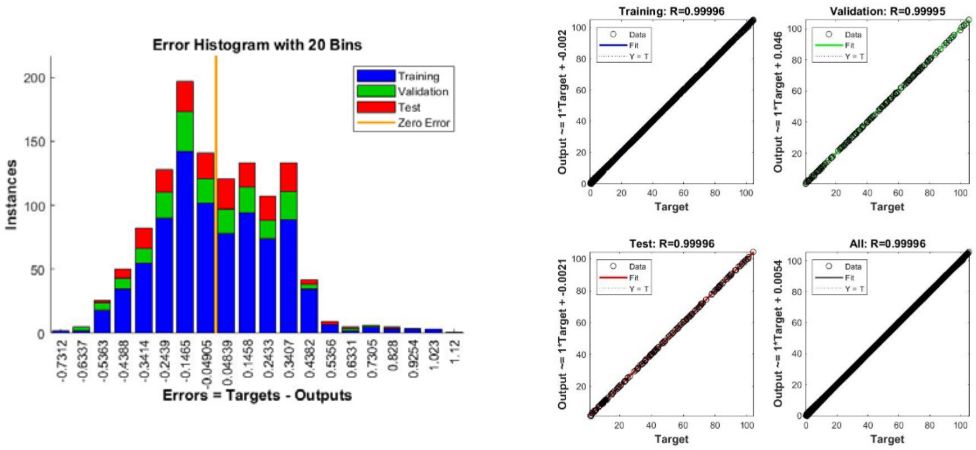

(c) **(d)**

Figure S4. Error histograms and regression plots of Pb (a,b) and Cd (c,d) ANN adsorption models generated with Levenberg–Marquardt algorithm.

Table S7. Comparison between calculated and ANN predicted values of the metal amount adsorbed onto **CHIT-PAAA.**

C_i (mg L^{-1})	q_e (mg g^{-1})	q_e ANN (mg g^{-1})	MSE
		**Pb**	
**10**	19.410	19.384	0.026
**20**	38.500	38.479	0.021
**40**	74.920	74.917	0.003
**60**	108.757	108.752	0.005
**80**	135.360	135.356	0.004
**100**	153.422	153.4173	0.0047
		**Cd**	
**10**	13.72	13.715	0.005
**20**	26.62	26.616	0.004
**40**	49.76	49.673	0.087
**60**	69.44	69.319	0.121
**80**	86.12	86.101	0.019
**100**	102.26	101.925	0.335

References

1. Langmuir, I. The Constitution and Fundamental Properties of Solids and Liquids. Part II.-Liquids. J. Franklin Inst. 1917, 184, 721.
2. Freundlich, H. Über Die Absorption in Lösungen. Zeitschrift für Phys. Chemie- Stöchiometrie und Verwandschaftslehre 1907, 57, 385–470.
3. Dubinin, M.M..; Radushkevich, L.V.. The Equation of the Characteristic Curve of Activated Charcoal. Proc. Acad. Sci. USSR Phys. Chem. Sect. 1947, 55, 331–337.
4. Temkin, M.J.; Pyzhev, V. Kinetics of Ammonia Synthesis on Promoted Iron Catalysts. Acta Physicochim. URSS 1940, 12, 217–222.
5. Redlich, O.; Peterson, D.L. A Useful Adsorption Isotherm. J. Phys. Chem. 1959, 63, 1024.
6. Sips, R. Combined Form of Langmuir and Freundlich Equations. J. Phys. Chem. 1948, 16, 490–495.
7. Toth, J. State Equation of the Solid Gas Interface Layer. Acta Chim. 1971, 69, 311–317.
8. Koble, R.A.; Corrigan, T.E. Adsorption Isotherms for Pure Hydrocarbons. Ind. Eng. Chem. 1952, 44, 383–387.
9. Khan, A.R.; Ataullah, R.; Al-Haddad, A. Equilibrium Adsorption Studies of Some Aromatic Pollutants from Dilute Aqueous Solutions on Activated Carbon at Different Temperatures. J. Colloid Interface Sci. 1994, 194, 154–165.
10. Lagergren, S.; Sven, K. Zur Theorie Der Sogennanten Adsorptiongeloster Stoffe. K. Sevenska Vetenskapsakademiens. Handl.

1898, 24, 1–39.
11. Ho, Y.S.; McKay, G. The Kinetics of Sorption of Divalent Metal Ions onto Sphagnum Moss Peat. Water Res. 2000, 34, 735–742.
12. Weber,W.J.; Morris, J.C. Kinetic of Adsorption on Carbon from Solution. Am. Soc. Civ. Eng. 1963, 89, 1–40.
13. Zeldowitsch, J. Über Den Mechanismus Der Katalytischen Oxidation Von CO a MnO2. URSS Acta Physiochim. 1934, 1, 364–449.
